# Transcriptomic, biochemical, and microbiome assessments into drought and salinity tolerance in durum wheat mediated by plant growth-promoting bacteria

**DOI:** 10.1007/s12298-025-01686-z

**Published:** 2025-11-19

**Authors:** Mohammad Yaghoubi Khanghahi, Hamada AbdElgawad, Maddalena Curci, Romain Garrigues, Shereen Magdy Korany, Emad A Alsherif, Erik Verbruggen, Matteo Spagnuolo, Rosangela Addesso, Adriano Sofo, Gerrit T.S. Beemster, Carmine Crecchio

**Affiliations:** 1 Department of Agricultural, Forestry, Food and Environmental Sciences (DAFE), Università degli Studi della Basilicata, Viale dell’Ateneo Lucano 10, 85100 Potenza, Italy; 2 Department of Soil, Plant and Food Sciences, University of Bari Aldo Moro, Via Amendola 165/A, 70126 Bari, Italy; 3 Department of Biology, Integrated Molecular Plant Physiology Research, University of Antwerp, 2020 Antwerp, Belgium; 4 Department of Botany and Microbiology, Faculty of Science, Beni-Suef University, Beni-Suef, 62511 Egypt; 5 Plants and Ecosystems Research Group, Department of Biology, University of Antwerp, Universiteitsplein 1C, 2610 Wilrijk, Belgium; 6 Department of Biology, College of Science, Princess Nourah bint Abdulrahman University, P.O. Box 84428, Riyadh, 11671 Saudi Arabia

**Keywords:** Durum wheat grain, Endophytic bacterial community, Plant growth-promoting bacteria, Stress, Transcriptomics

## Abstract

This study investigates the efficacy of plant growth-promoting bacteria (PGPB) in improving stress tolerance in plants by analyzing the molecular and biochemical bases in durum wheat grain. An experiment was conducted where soil and seeds were inoculated with PGPB, under drought and salinity stress. 16 S rRNA sequencing indicated no change in grain bacterial communities in response to biofertilizers and stress. However, a genome-wide analysis identified 153 up-regulated and 33 down-regulated plant genes in response to PGPB, predominantly enriched in stress-related biological processes. These genes specifically encode for proteins involved in metabolite interconversion enzyme, chaperone, protein modifying enzyme, and transporters, which are functionally related groups assisting protein folding in the cell under stress conditions. Moreover, pathway analysis confirmed related changes at the metabolite and enzyme activity levels. In this regard, PGPB-treated plants exhibited heightened activity of both enzymatic and non-enzymatic (e.g., thioredoxins, peroxiredoxins, etc.) antioxidants under stress, showcasing significant enhancements ranging from + 27% to + 283% and + 36% to + 266%, respectively. Further elucidation of biochemical pathways revealed alterations in the activation of non-antioxidant enzymes in PGPB-treated plants, exemplified by increased activities of glutamate synthase (40–44%) and decreased activities of protein-tyrosine-phosphatase (29–31%) under both stresses, as well as elevated activities of anthocyanidin reductase (91%) and lipoxygenases (18%) specifically under drought. Overall, the present research highlighted the potential of beneficial bacteria in improving plant stress tolerance, especially under drought, through shifting transcriptome expression of plant genes and employing multiple protective strategies which can complement each other.

**Supplementary Information:**

The online version contains supplementary material available at 10.1007/s12298-025-01686-z.

## Introduction

Plant growth-promoting bacteria (PGPB) are a critical component in customizing the microbiomes for sustainable crop production, and play a key role in promoting plant growth, controlling pathogens, and improving plant stress tolerance (Arif et al. 2020; Chen et al. 2022). There seems to be some evidence to indicate that inoculation of soil, seed, or plant with beneficial microorganisms can regulate plant microbiome structure and function (dos Santos and Olivares 2022). These findings led to microbiome engineering, as an emerging biotechnological strategy, which could improve plant growth by manipulating the plant/microbe holobiont (Aref et al. 2020).

Researchers have coined the term “symbiosis cascade effect” to describe the mutualistic relationship between plants and microbes, wherein symbionts and their host plant collectively shape the plant microbiome (Uroz et al. 2019). Within this context, grain microbiota has been observed to transmit either horizontally, acquired from the surrounding environment, or vertically, inherited from the parent (Shade et al. 2017; Addesso et al. 2022). Consequently, it can be inferred that alterations in the grain microbiome, influenced by environmental stress and microbial inoculation, may not only impact the current generation but also be inherited by subsequent generations of plants, potentially conferring benefits to them (Shade et al. 2017). As a result, concerns have been raised regarding the safety of employing beneficial bacteria and the extent to which they can alter the grain microbiome and/or holobiont structure. Understanding how the grain microbiome responds to PGPB inoculation is crucial for establishing the reliability of its application as a biofertilizer, particularly since certain changes in the grain microbiome may pose risks to both crops and human health (Gross 2022; Yaghoubi et al. 2021a). The signal transition from the environment is the main mechanism adopted by plants to regulate genes in response to stress, which eventually can result in triggering the accumulation of transcription factors and in activating gene expression (Thiebaut et al. 2019). Recent trends in gene expression patterns have also led to a proliferation of studies indicating they have been regulated by epigenetic states when plants are exposed to stimuli from PGPB and abiotic stresses, thereby eventually affecting crop production (Chen et al. 2022). Moreover, some gene expression alterations and stress-induced epigenetic modifications can be heritable to the next generations and become epigenetic memories in the process of plant adaptation to stress (Thiebaut et al. 2019; Chen et al. 2022). Nevertheless, little is known about epigenetic modifications in PGPB-mediated plants, and it is not clear how beneficial bacteria affect the expression of plant genes (Chen et al. 2022), especially compared to plants treated with traditional chemical fertilizers.

The debate about the effectiveness of PGPB has gained fresh prominence regarding their colonizing capability to compete with the endophytic microbiota under environmental changes. Although there have been many longitudinal studies on the agronomic and physiological traits of PGPB-treated plants, much remains to be investigated about the monitoring of PGPB in plants, in particular, about specific shifts in endophytic bacterial communities and transcriptional responses of plant genes (Gross 2022; Yaghoubi et al. 2021a). The present research aimed to investigate how fertilization by a consortium of PGPB strains affects the bacterial community inhabiting durum wheat (*Triticum durum*) grain in non-stress and stress (drought and salinity) conditions. A genome-wide transcriptome analysis was also used to provide considerable information on highly and differentially expressed genes involved in plant stress tolerance. Therefore, this research sought to address the following questions: (i) are the grain bacterial communities and gene expression pattern of the durum wheat plant altered by applying a bacterial consortium of PGPB strains under stress and non-stress conditions? and (ii) what molecular mechanisms underlie mitigating drought/salinity stress in PGPB-treated plants?

The present research is a continuation of a comprehensive research project aiming at advancing the understanding of the relationship between soil biological fertility and plant and soil microbiome. As part of this project, several beneficial bacterial strains have been isolated from durum wheat fields in southern Italy (Basilicata region) and characterized (Yaghoubi et al. 2021b). Among these strains, *Acinetobacter pittii* (DSM 21653 ^T^), *Acinetobacter oleivorans* (DR1^T^), *Acinetobacter calcoaceticus* (NCCB 22016 ^T^), and *Comamonas testosteroni* (KS 0043 ^T^) emerged as the most promising by showing promise of favorable dissolution of insoluble complexes of phosphate (115.5 mg l^− 1^), potassium (35.6 mg l^− 1^), and zinc (389.90 mg l^− 1^), as well as biological fixation of nitrogen (25.2 mg l^− 1^) in vitro conditions, respectively (Yaghoubi et al. 2021b). The potential of these beneficial microorganisms, as PGPB, was then demonstrated in a greenhouse experiment by improving grain yield and photosynthesis efficiency under stress (Yaghoubi et al. 2021c). The application of these PGPB as a bio-inoculant led to more pronounced alterations in the bacterial communities residing within the roots compared to those present in the soil rhizosphere under both control and stress conditions (Yaghoubi et al. 2021a). In addition, PGPB improved tissue chemical composition and nutritional values of durum wheat grains to a comparable degree as chemical fertilizers, especially under stressful conditions, highlighting the efficacy of PGPB as a sustainable fertilization approach (Yaghoubi et al. 2022). Although the growth, physiological and biochemical bases are well study, the molecular mechanisms underlying the mitigating drought/salinity stress in PGPB-treated plants are not well understood. Thus, this study aimed to explore molecular and biochemical bases of improved production of durum wheat grain under drought and salinity stress conditions.

## Materials and methods

### Greenhouse experiment

The present experiment was run by the same experimental setup explained in the previously mentioned experiments (Yaghoubi et al. 2020; 2021a; 2021b; 2021c; 2022) but dealing with different sets of data. Accordingly, the experiment was based on two fertilization and stress treatments, the first of which consisted of two levels, (i) no fertilization (Co) and (ii) biofertilization (BC) by seed inoculation with the biofertilizer consortium of four PGPB strains and pot inoculation by the bacterial suspension every three weeks. In short, a cross-streak assessment between co-inoculated strains was conducted prior to employing a consortium comprising four PGPB strains (Yaghoubi et al. 2021c). The absence of inhibition zones or antagonistic symptoms in the plates indicated the compatibility of the PGPB strains. Then, the inoculants were cultured in Nutrient Broth (NB) medium, initiated from a 24-hour bacterial culture on a Nutrient Agar (NA) plate, and grown under agitation at 100 rpm for 24 h at 29 °C. Following centrifugation at 5000 rpm for 5 min, the concentrated bacterial culture pellets were washed with a sterile solution of 0.9% w/v potassium chloride. The optical density of the bacterial suspension at 600 nm was adjusted to 0.6–0.7, corresponding to a count of 10^6^ CFU ml^− 1^.

Stress treatment was defined at three levels: (i) non-stress, (ii) drought stress at 40% of field capacity (achieved by reduced watering), and (iii) salinity stress at 150 mM NaCl, reached by applying saline solutions every three days starting from the 63rd day after sowing (DAS), corresponding to the booting stage, until the 81st DAS, corresponding to the heading stage. The levels of applied stress treatments were chosen based on preliminary experiments, in which the grain yield in plants under stress was almost half of that in control conditions (Yaghoubi et al. 2021b).

The pot experiment followed a completely randomized design using unsterilized clay loam soil (Eutri-Fluvic Cambisols), collected from the same fields where the PGPB strains had been isolated. The soil had 0.99% organic C, 270 µg C g^− 1^ microbial biomass C, a C: N ratio of 10.4, 31% water content at field capacity (− 0.3 MPa), 17% wilting point (− 15 MPa), an electrical conductivity of 0.44 dS m^− 1^ in the soil saturation extract (EC), and a pH of 7.5. Plants were cultivated in a greenhouse under constant light/temperature conditions (20 °C, 14 h light, and 10 h dark). Additionally, pots were rotated weekly to minimize edge effects. Grain samples were harvested from each of the 36 pots (representing two fertilization treatments, three stress levels, and six replications) at 124th DAS. These samples were promptly collected in liquid nitrogen and stored at -80 °C for subsequent biochemical and molecular analyses.

## DNA extraction and 16s rRNA sequencing

DNA extraction of ground grain samples was carried out using the soil DNA extraction kit (MP Biomedicals™ FastDNA™ SPIN Kit, Santa Ana, CA, USA), and quantified using the Nano-Drop spectrophotometer (ND-1000, EuroClone, Milan, Italy). Aliquot of the extracted DNA was subjected to PCR with the 341F (5’– CCTACGGGNGGCWGCAG − 3’) and 805R (5’– GACTACHVGGGTATCTAATCC – 3’) primer set targeting V3 and V4 hypervariable region of the 16 S rRNA. PCR products were diluted (60X) and used for the second PCR round after adding the dual-index barcoded primers with Illumina sequencing adapters to the amplicon target. The library was prepared by combining the PCR products, then quantified by qPCR and sequenced on the Illumina MiSeq platform (Illumina, San Diego, CA) at IGA Technology Service (Udine, Italy). Metagenomic Rast Server (MG-RAST) (http://metagenomics.anl.gov) was used for processing raw reads (Meyer et al. 2008). The sequences were clustered into operational taxonomical units (OTUs) at 97% similarity and then subjected to taxonomic assignments using the Ribosomal Database Project (RDP) Naïve Bayesian classifier.

## RNA extraction and sequencing analysis

RNA extraction from grains was carried out according to the method described by Li and Trick (2005) with some modifications. Briefly, a 500 µL extraction *buffer I* (100 mM Tris (pH 8.0), 50 mM EDTA, 150 mM LiCl, 1.5% sodium dodecyl sulfate (SDS), 1.5% 2-mercaptoethanol) aliquot was added to the ground grain samples (150 mg), followed by mixing by vortex and incubating on ice for 10 min. 250 µL phenol: chloroform: isoamyl alcohol (25:24:1, v/v) was added, mixed by inversion and centrifuged at 14,000 rpm for 15 min at 4 °C. 250 µL extraction *buffer II* (70% guanidine thiocyanate (w/v), 0.75 M sodium citrate, 10% lauryl-sarcosine, 2 M sodium acetate) were added to the supernatant, mixed by inversion and incubated at room temperature for 15 min. After the addition of 250 µL of chloroform: isoamyl alcohol (24:1, v/v), mixing by inversion and centrifugation (14,000 rpm, 15 min, at 4 °C), the upper phase was transferred to the new tubes containing 300 µL isopropanol and 250 µL 1.2 M sodium chloride. The samples were mixed by inversion, incubated on ice for 15 min, and centrifuged at 14,000 rpm for 15 min at 4 °C. The pellet was washed with 400 µL ethanol (70% v/v), centrifuged at 8,000 rpm for 5 min and dried at room temperature for 15 min. Finally, the RNA pellet was re-suspended in 50 µL RNase-free water. The reverse transcription into cDNA and library preparation were conducted using total RNA samples (RIN > 7) and sequenced on the Illumina^®^ HiSeq platform (2 × 250 bp) at the Novogene Co., Ltd. (Cambridge, UK, https://www.novogene.com).

The sequencing data were analyzed on the Galaxy web server (https://usegalaxy.eu), and then quality control of Fastq files was performed by FastQC (Batut et al. 2022). Subsequently, to eliminate low-quality reads, the data were trimmed with Trimmomatic v0.39 (Bolger et al. 2014), employing parameters (LEADING:20 TRAILING:20 ILLUMINACLIP:$adapters:2:40:15 SLIDINGWINDOW:4:20 MINLEN:36). For mapping and alignment, the latest durum wheat (*T. turgidum* cv. Svevo Rel. 1.0 genome assembly, 2019) reference genome (Maccaferriet al. 2019) was obtained from NCBI’s database. The RNA-seq data underwent alignment to the reference genome via STAR (v.2.7.2b) alignment software (Dobin et al. 2013). The genome indexing command generated a genome index file with option --genomeChrBinNbits 7. Both the index file and the samples were then mapped to the reference genome using STAR’s default settings. Subsequently, a Python script was employed to merge the sample count files, facilitating downstream analysis for differential expression across all samples. After read counting, the expression dataset was also normalized and analyzed using the DeSeq2 package to calculate differentially expressed genes (filtered by a 5% false discovery rate (FDR), *P*-value < 0.05, according to Love et al. (2014). A principal component analysis (PCA), as a statistical procedure for summarizing the information content of a large data set, was performed to explain the biological variations among samples visually. In this regard, PCA was conducted on the entire transcriptome, considering all expressed genes, regardless of their expression levels or patterns. This comprehensive approach allowed the visualization of the overall biological variations between the samples, facilitating a clear understanding of the influence of stress treatments and bacterial inoculation on gene expression in durum wheat grains. Furthermore, the Panther classification system of the gene ontology consortium (http://geneontology.org) was also utilized for plant GOSlim classification and ascertaining the ontological relevance of differentially expressed genes adjusted by an FDR correction and Fisher test-type (Mi et al. 2019).

## Assessment of MDA content

Malondialdehyde (MDA) was extracted from 50 mg of ground grain samples that were homogenized with 1 ml of 80% v/v ethanol using a MagNA Lyser (Roche, Vilvoorde, Belgium). Then, the mixture was centrifuged at 12,000 rpm for 10 min at 4 °C to obtain a clear supernatant. The MDA content was analyzed using a thiobarbituric acid (TBA) assay; to each 200 µl of the extracted supernatant, 800 µl of TBA solution (0.67% w/v in 20% acetic acid) was added. This mixture was then incubated at 95 °C for 30 min to facilitate the formation of the MDA-TBA adduct. After cooling, the absorbance was measured at 532 nm using a microplate reader, while absorbance at 600 nm was also recorded to account for nonspecific readings. The standard curve was generated from known MDA concentrations of 0, 2, 5, 10, 20, and 30 µmol/L; these were bench-marked alongside the samples during testing to relate the absorbance values to MDA concentrations. The final MDA concentrations in the samples were expressed as nmol per gram (AbdElgawad et al. 2016).

## Assessment of antioxidant enzyme activities

Antioxidants enzymes of durum wheat grains were extracted by homogenizing ground grain samples in 0.05 M potassium-phosphate buffer (pH 7.8) and centrifuged at 10,000 × g for 22 min at 4 °C. The collected supernatant was used for the assessment of different antioxidant enzyme activities (Nawaz et al. 2020). Accordingly, superoxide dismutase (SOD) activity was measured by monitoring the inhibition of 50% of the nitro-blue tetrazolium (NBT) reduction at 560 nm (Giannoplitis and Ries 1977). The decrease in absorbance at 240 and 470 nm was read to measure the catalase (CAT) and peroxidase (POD) activity based on the hydrogen peroxide (H_2_O_2_) decomposition and pyrogallol oxidation, respectively (Chance and Maehly 1995). A decrease of 1 mM (w/v) ascorbic acid at 290 nm was considered to measure ascorbate peroxidase (APX) activity (Nakano and Asada 1981). Formation of ascorbate and oxidation of reduced nicotinamide adenine dinucleotide (NADH) was monitored spectrophotometrically at 265 and 340 nm to measure dehydroascorbate reductase (DHAR) (Dalton et al. 1986) and monodehydroascorbate reductase (MDHAR) (Murshed et al. 2008) activities, respectively. The activity of glutathione reductase (GR) and glutathione peroxidase (GPx) was determined spectrophotometrically at 340 nm based on NADH oxidation and reduction, respectively (Cakmak et al. 1993; Elia et al. 2003). Decrease in H_2_O_2_ and 5,5′-dithiobis(2-nitrobenzoic acid) (DTNB) at 380 and 412 nm was considered to measure peroxiredoxins (Prx) and thioredoxin (Trx) based on the protocols described by Horling et al. (2003) and Pascual et al. (2011), respectively. Glutaredoxin (Grx) molecule content was assessed by NADPH-dependent reduction of 2-hydroxyethyl disulfide (HED) in the presence of GR at 340 nm (Holmgren 1989).

Lipoxygenase activity was determined based on the modified ferrous oxidation-xylenol orange method, spectrophotometrically measured at 560 nm (Timabud et al. 2013). Estimation of ferredoxin-NADP(H) reductase (Frx) was carried out according to the method described by Zannetti and Curti (1980) through the reduction of K_3_Fe(CN)_6_ by NADPH in the presence of a regenerating system. A modification of the pH differential method was used for the determination of anthocyanin content in grain samples (Zhang et al. 2012). Anthocyanidin reductase (ANR) activity was measured by epicatechin content, as described by Wen et al. (2012).

Protein tyrosine phosphatase (PTP) activity was evaluated, as it is vital for the present study and can shed light on the regulatory mechanisms of signaling pathways in durum wheat grains under both normal and stress conditions. To measure PTP activity, we prepared homogenates from durum wheat grains to extract soluble and particulate proteins without sodium orthovanadate. The synthetic phosphopeptide was incubated with varying concentrations of these protein extracts to assess PTP activity. This enzymatic response was enhanced by adding 5 mM β-mercaptoethanol. To confirm the presence of PTP, we employed sodium orthovanadate, a known inhibitor of tyrosine phosphatases, and assessed PTP activity across increasing concentrations of this inhibitor to generate a dose-response curve that indicated the degree of phosphatase activity inhibition (Rodríguez-Zapata and Hernández-Sotomayor 1998).

### Assessment of carbohydrates and carbohydrate metabolism enzyme activities

The total sugars and the reducing sugars were determined separately based on the phenol sulphuric acid and dinitro salicylic acid methods, respectively, and then the content of non-reducing sugars was computed by subtracting reducing sugars from the total sugars (Miller 1959; Kumari and Asthir 2016). Starch content was also evaluated by anthrone method (Yemm and Willis 1954). Photometric o-toluidine aldose back-measurement was used to determine the aldose reductase (AR) activity (Matkovics and Szabó 1977). Measurement of β-amylase activity was done using a protocol of McCleary and Codd (1989) by recording the p-nitrophenol released from the substrate, at 410 nm. The inhibition activity of Xylanase inhibitor (XI) was assessed based on the colourimetric Xylazyme-AX method (Fierens et al. 2007). Uridine diphosphate (UDP) glycosyltransferases (UGTs) assay is fully described by Srivastava et al. (2021). According to Palmgren (1990), a decrease in the absorbance at 340 nm by coupling ATP hydrolysis enzymatically to the oxidation of NADH was evaluated to measure ATPase activity. Chitinase activity was also assessed colourimetrically as defined based on the rate of hydrolysis of colloidal chitin into N-acetylglucosamine (Reissig et al. 1955).

## Germination test

The harvested durum wheat grains were subjected to sterilization by soaking in a sodium hypochlorite (NaOCl) solution (35% v/v) for 15 min, followed by rinsing with sterile distilled water. Twenty-five wheat seeds were then evenly distributed onto two layers of filter paper in Petri dishes (9 cm in diameter), following the ISTA (2018) guidelines, with three replicates. Each Petri dish was filled with 7 ml of distilled water containing 0.1% thiram fungicide. Seed germination was initiated in darkness at a temperature of 25 °C and monitored daily for 7 days. Germination was considered complete when the radicle reached half the length of the seed. Germination parameters, including seed germination energy (GE), germination percentage (GP), germination index (GI), vitality index (VI), and simplified vitality index (SVI), were determined according to the method described by Wu et al. (2024), as follows:

- GE = (number of germinated seeds on the 3rd day / total seeds) × 100%
- GP= (number of germinated seeds on the 7th day / total seeds) × 100%.
- GI = ∑ (the number of germinations per day / the number of days corresponding).
- VI = GI × the seedling length (cm) at the end of germination (L).
- SVI = GP × L

### Statistics

Transcriptome experiments were performed for the new greenhouse experiment with three independent biological samples to ensure statistical robustness (*n* = 3). As the test of homogeneity of variances (Bartlett) indicated that the dependent variables of biochemical analysis had equal variances across two new and previous pot experiments, the results of metabolomics assessment were analyzed as a single experiment with six replicates. The data were presented as mean ± standard deviation (SD) and analyzed using IBM SPSS Statistics (V21.0; SPSS^®^ Inc., Chicago, IL, USA). Mean comparisons were conducted using post-hoc Tukey**’**s Honestly Significant Difference (HSD) test at 0.05 probability level.

## Results

### Grain endophytic bacterial community

After subjecting plants to several treatments, the harvested grains were analyzed to investigate the bacterial community and reveal the effective behavior of PGPB on bacterial colonization. Accordingly, the most abundant phyla in all grain samples were *Bacteroidota*, ranging from 72.7% to 75.9% of the total sequences, followed by *Proteobacteria* (19.2–20.3%), *Myxococcota* (2.6–3.1%), and *Acidobacteriota* (1.4–1.6%). Five abundant orders with a relative abundance greater than 2% were detected, namely *Chitinophagales* (43.2–47%), *Cytophagales* (33.4–37.4%), *Polyangiales* (4.6–5.3%), *Caulobacterales* (6.3–7%), and *Rhizobiales* (2.5–3%) were more present in the samples as compared to other taxa (Table 1). Moreover, the relative abundances of sequences from genera *Acinetobacter* and *Comamonas*, used as bioinoculants in the present research, were very low in all samples (Supplementary Table 1). However, neither fertilizer nor stress treatments had a significant effect on wheat grain bacterial communities. In addition, the absence of significant differences in diversity indices (Pielou’s Evenness and Shannon entropy) indicated no difference in bacterial alpha diversity in response to fertilization or stress treatments (Supplementary Fig. S1).

**Table 1:** Relative abundance of (A) bacterial phyla (relative abundance > 1%) and (B) orders (relative abundance > 2%) of durum wheat grain as affected by biofertilizer consortium under optimal and stress conditions

(A)	Non-stress		Drought		Salinity	
Phylum	Co	BC	Co	BC	Co	BC
Bacteroidota	73.86 ± 1.78 a	75.67 ± 2.70 a	73.84 ± 2.20 a	74.55 ± 1.10 a	75.95 ± 1.11 a	72.66 ± 2.53 a
Proteobacteria	19.24 ± 0.58 a	19.59 ± 0.65 a	20.31 ± 0.23 a	19.44 ± 0.60 a	19.64 ± 0.68 a	19.88 ± 0.66 a
Myxococcota	2.80 ± 0. 14 a	2.62 ± 0.21 a	2.65 ± 0.18 a	3.02 ± 0. 56 a	3.10 ± 0.44 a	2.88 ± 0.17 a
Acidobacteriota	1.54 ± 0.12 a	1.63 ± 0.17 a	1.60 ± 0.07 a	1.39 ± 0.11 a	1.40 ± 0.10 a	1.48 ± 0.23 a
*(B)* Order						
*Chitinophagales*	43.19 ± 1.08 a	46.99 ± 2.17 a	43.00 ± 1.25 a	43.98 ± 1.29 a	44.35 ± 3.01 a	43.44 ± 4.41 a
*Cytophagales*	34.69 ± 2.44 a	33.45 ± 1.84 a	35.28 ± 1.08 a	34.67 ± 1.86 a	37.43 ± 3.06 a	34.13 ± 1.63 a
*Polyangiales*	5.29 ± 0.25 a	4.74 ± 0.24 a	4.63 ± 0.35 a	4.76 ± 0.53 a	4.96 ± 0.43 a	5.24 ± 0.65 a
*Caulobacterales*	6.34 ± 0.50 a	7.01 ± 0.51 a	6.97 ± 0.31 a	6.32 ± 0.54 a	6.65 ± 0.29 a	6.31 ± 0.32 a
*Rhizobiales*	2.77 ± 0.28 a	2.92 ± 0.14 a	3.03 ± 0.21 a	2.54 ± 0.22 a	2.55 ± 0.19 a	2.92 ± 0.34 a

Means (± standard deviation; *n* = 3) in each phylum and order followed by similar letter(s) are not significantly different at 5% probability level (Tukey test).

Co, No fertilization (control); BC, Biofertilizer consortium of four PGPB strains.

### Biofertilizers induce specific transcriptional responses under stress conditions

**Fig. 1:**
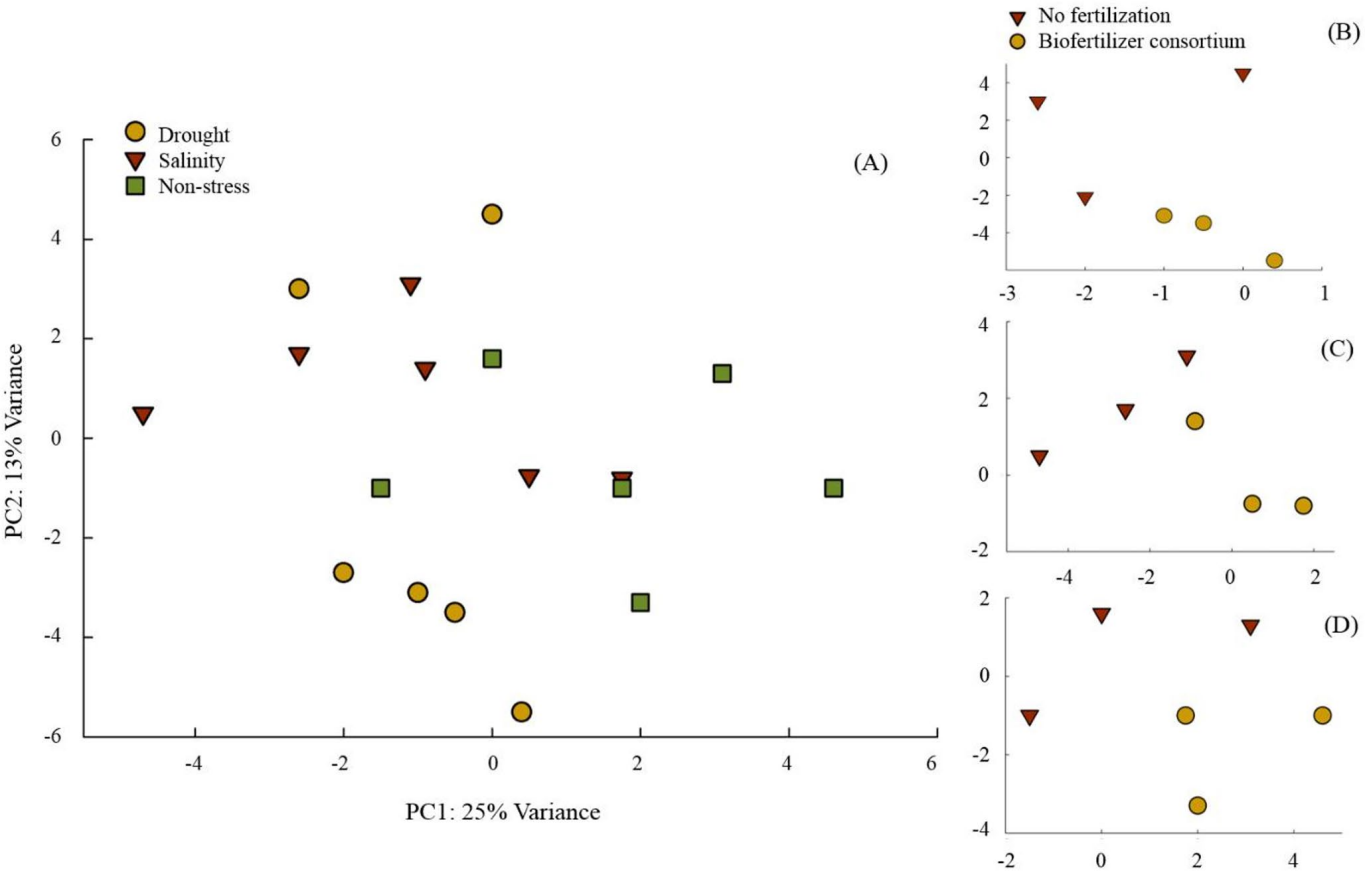
The effect of stress treatments (**A**) and inoculation with a consortium of four beneficial bacteria under drought (**B**), salinity (**C**) and non-stress (**D**) on the biological variations of expressed genes among samples in wheat grain based on a principal component analysis (PCA). The PCA, conducted on all sequenced genes across three biological replicates per treatment, was performed to independently evaluate their influence on gene expression variation, regardless of expression levels or patterns

A genome-wide transcriptome analysis indicated that approximately 0.3% of the ~ 63,000 sequenced genes in the grains were significantly up- or down-regulated in response to the interaction effects of biofertilization and stress treatments (FDR < 0.05 and log2_FC_ > 0.5). A principal component analysis (PCA) plot was used to visualize patterns of similarities among identified genes under different treatments (Fig. 1). Although no differences were observed among stress treatments in terms of groups of all sequenced genes in the samples (Fig. 1A), such genes do separate clearly in response to the biofertilizer consortium within each stress level (Fig. 1B, C, D).

**Fig. 2:**
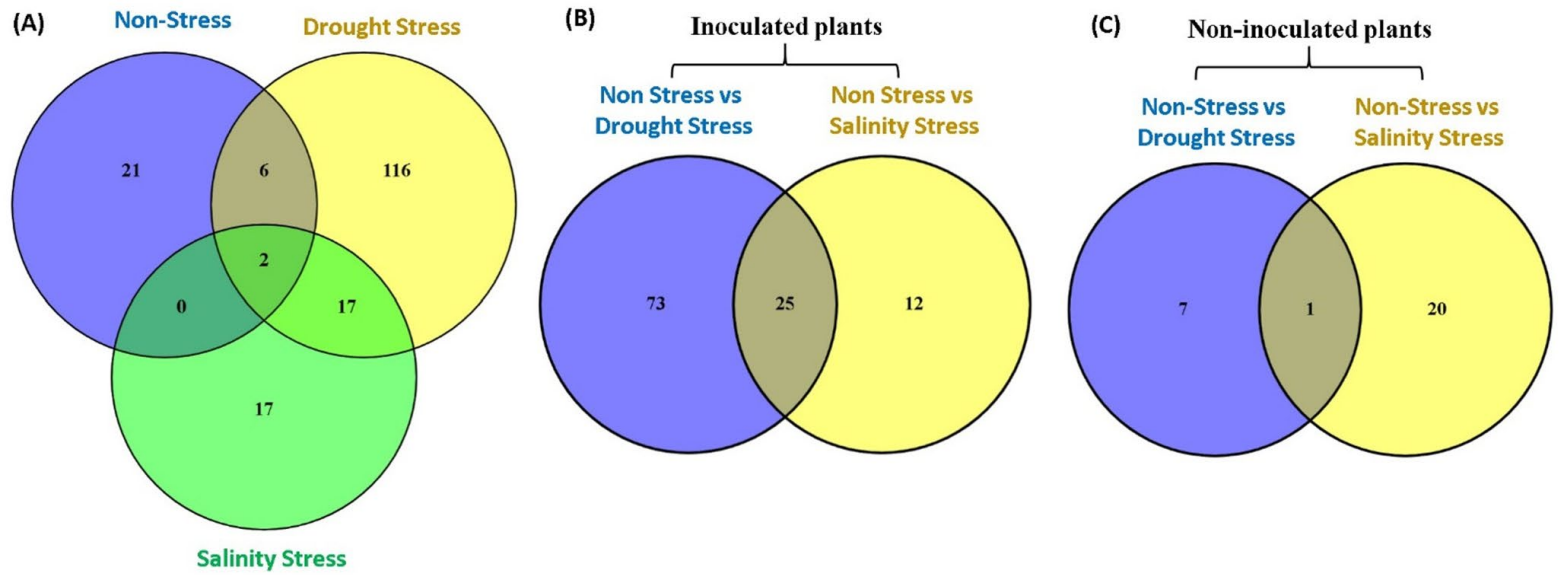
Venn diagrams illustrating the differential expression of genes (DEGs) in biofertilizer-treated plants under various stress conditions. Panel (**A**) compares gene expression profiles in non-stress, drought stress, and salinity stress, displaying 29, 141, and 36 DEGs, respectively, in inoculated plants compared to non-inoculated, with analyses performed on samples from three replicates per treatment. The overlap indicates the presence of common genes among treatments. Further analysis of DEGs between non-stress/drought stress and non-stress/salinity stress in inoculated plants revealed specific gene expressions linked to biofertilizer treatment and stress responses (**B**). Panel (**C**) compares DEGs under non-stress versus drought stress and non-stress versus salinity stress in non-inoculated plants, also based on three replicates per treatment. Genes exhibiting significant differential expression were identified with a false discovery rate (FDR) of < 0.05

Results revealed significant alterations in gene expression profiles, with 29, 141, and 36 genes showing differential expression (FDR < 0.05) in biofertilizer-treated plants compared to control plants under non-stress, drought, and salinity stress, respectively (Fig. 2A; Supplementary Table 2). Furthermore, a comparative analysis between inoculated and non-inoculated plants highlighted distinct responses to stress. Specifically, 98 and 37 genes exhibited differential expression (FDR < 0.05) in biofertilizer-inoculated plants under drought and salinity stress, respectively, compared to non-stress conditions (Fig. 2B; Supplementary Table 2). In contrast, non-inoculated plants displayed fewer changes, with only 21 and 8 genes showing altered expression (FDR < 0.05) under drought and salinity stress, respectively (Fig. 2C; Supplementary Table 2).

Under non-stress conditions, grains from plants inoculated with PGPB exhibit upregulation of some genes involved in grain filling and maturation processes, including those encoding for storage proteins (e.g., cupincin-like, Vicilin-like seed storage protein), germination process (e.g., oleosin 18 kDa-like), and grain quality traits (e.g., gamma gliadin). Moreover, genes related to nutrient uptake and metabolism, such as amino acid transporters and micronutrient transporters (e.g., glycerophosphodiester phosphodiesterase GDPD2-like, lysosomal amino acid transporter, organic cation/carnitine transporter 4-like), show enhanced expression levels in grains of PGPR-inoculated plants compared to control plants. Genes associated with stress response pathways (e.g., aldose reductase, late embryogenesis abundant proteins, glycine-rich RNA-binding protein, luminal-binding protein 2) are also upregulated in PGPB-treated plants under non-stress (Supplementary Table 2).

Nevertheless, the present research delved into the interaction effects between biofertilization and stress, as a higher number of differentially expressed genes under these combined conditions was observed. Accordingly, 186 genes showed more than twofold significant changes in expression (FDR < 0.05 and log2_FC_ > 0.5) under the biofertilizer consortium treatment under stress, especially in plants treated by drought stress, and consequently were classified into two groups: up-regulated genes and down-regulated genes, based on their expression patterns.

**Fig. 3:**
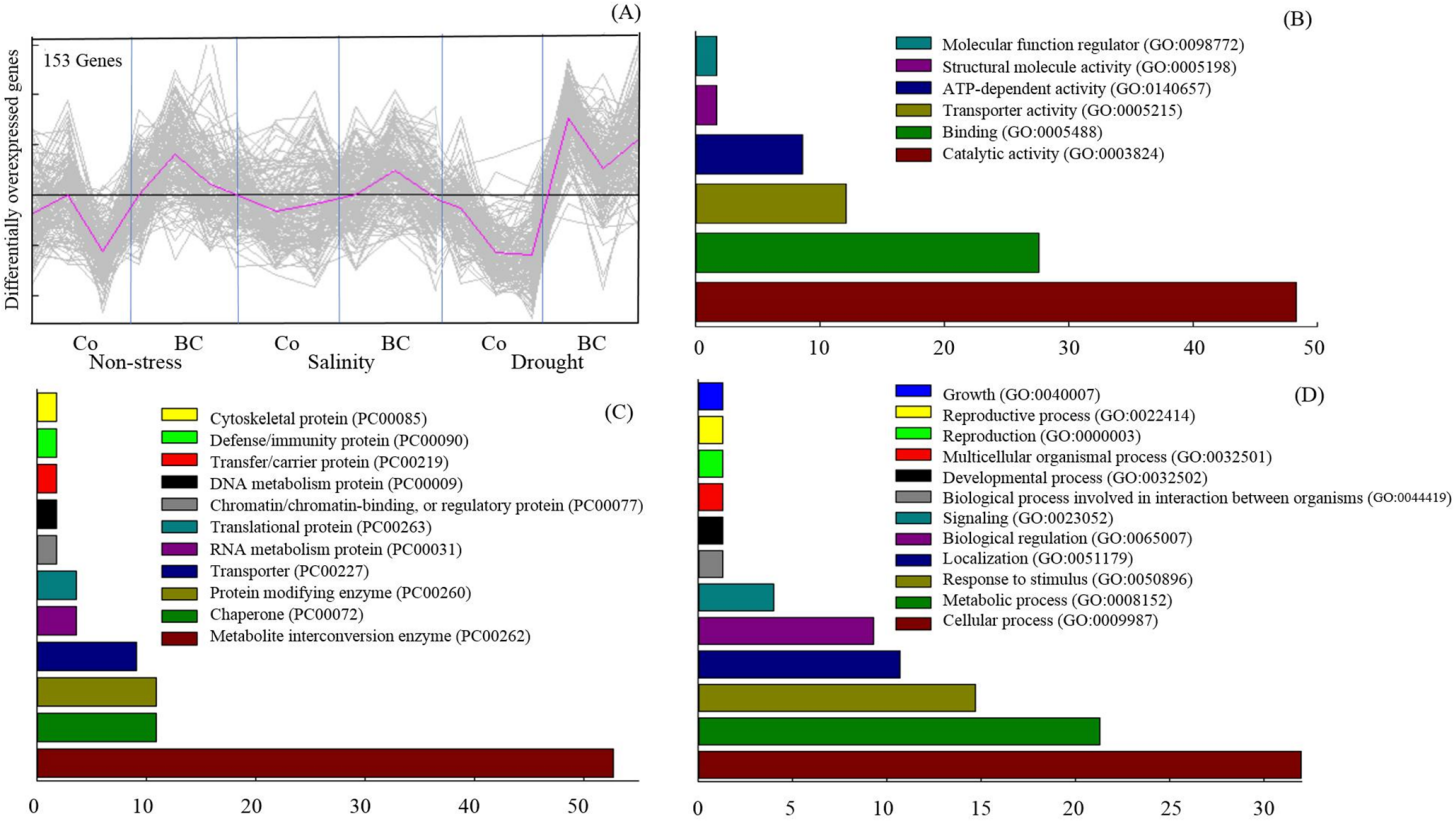
The effect of inoculation with a consortium of four beneficial bacteria and stress treatments on up-regulated genes (**A**) and their molecular function (**B**), protein class (**C**), and biological process classifications (**D**) according to Panther classification system in wheat grain. Co, Control (No fertilization); BC, Biofertilizer consortium

The plant GOSlim classification, which is a simple version of Gene Ontology, predicted the largest cluster contained 153 genes, whose expressions were increased in plants treated with biofertilizers (Fig. 3). By focusing on the molecular function category of this group of genes, we found a number of genes which were assigned to “catalytic activity” (48.3%), followed by “function of DNA binding” (27.6%), “transporter activity” (12.2%), and “ATP-dependent activity” (8.6%) (Fig. 3B). In terms of the protein classes, they mostly belonged to the “metabolite interconversion enzyme” (52%), “chaperone” (10.9%), “protein modifying enzyme” (10.9%), and “transporter” (9.1%) ones (Fig. 3C). According to the biological processes, most genes of this group were involved in “cellular process” (32%), “metabolic process” (21.3%), “response to stimulus” (14.7%), “localization” (10.7%), “biological regulation” (9.3%), and “signaling” (4%) ones (Fig. 3D).

**Fig. 4:**
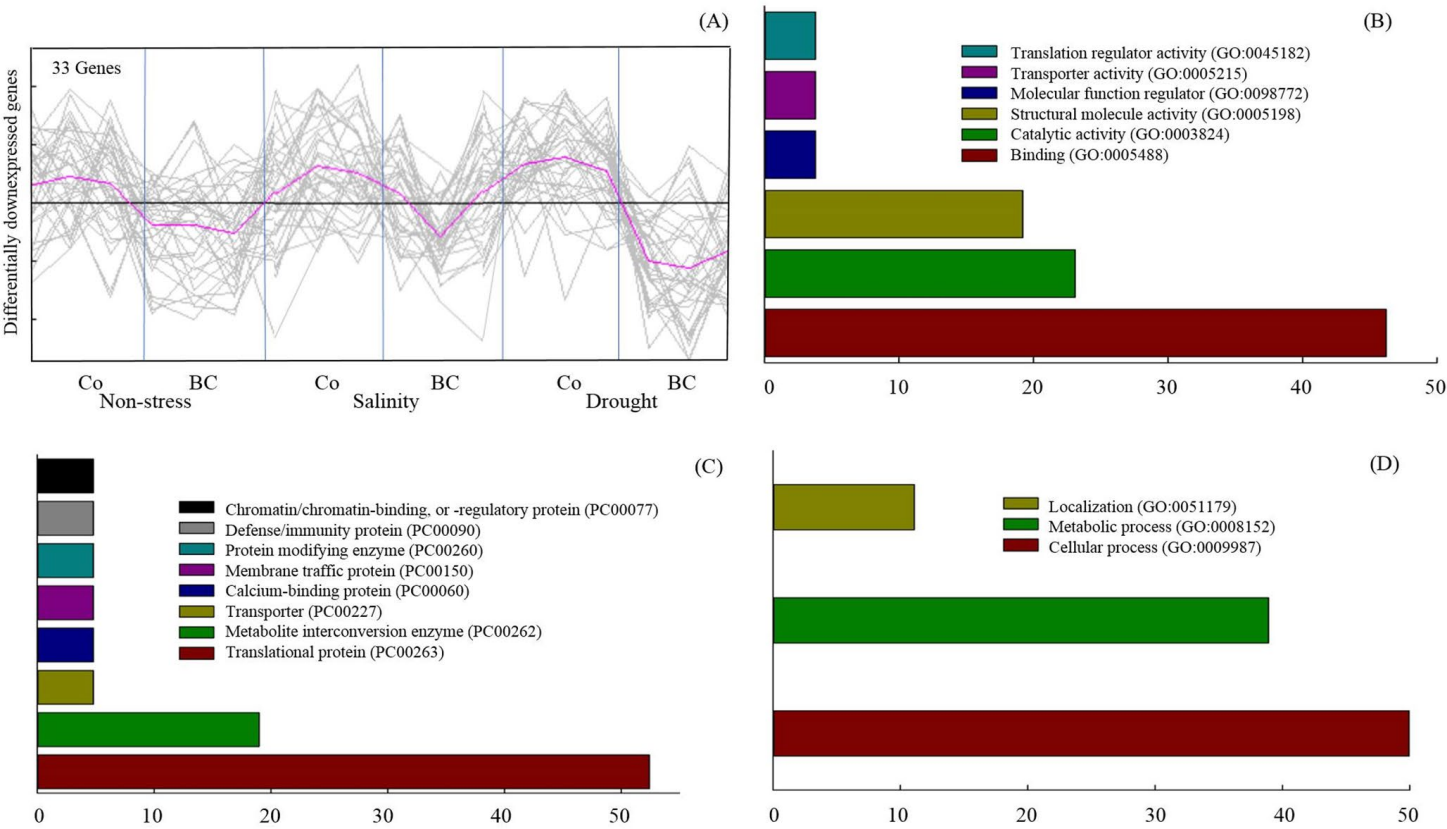
The effect of inoculation with a consortium of four beneficial bacteria and stress treatments on down-regulated genes (**A**) and their classification based on molecular function (**B**), protein class (**C**), and biological process (**D**) according to Panther classification system in wheat grain. Co: Control (No fertilization); BC: Biofertilizer consortium

Also, 33 genes, whose expression was significantly decreased by biofertilizer treatment, were analyzed (Fig. 4). Accordingly, genes involved in “function of DNA binding” (46.2%), “catalytic activity” (23.1%), and “structural molecule activity” (19.2%) were pronounced in the molecular function category (Fig. 4B). In terms of the protein class, these genes were classified within the two majority categories including “translational protein” (52.4%), and “metabolite interconversion enzyme” (19%) (Fig. 4C). This group of genes was also categorized on the basis of the biological process, in which 50, 38.9 and 11.1% of them belonged to “cellular process”, “metabolic process”, and “localization”, respectively (Fig. 4D).

Among all the annotation classifications, the biological process constituted the central focus of our study, in which we could better realize the general response of plants to the biofertilizer under stress conditions using a statistical overrepresentation test (Tables 2 and 3). Accordingly, the genes involved in response to stress (e.g. the Panther family/subfamily of catalase isozyme A, catalase isozyme B, peroxygenase, glutathione s-transferase 1, water stress-inducible protein RAB21, salt-induced YSK2 dehydrin 2 (DHN2), small heat shock domain-containing protein, aldehyde oxygenase, alcohol dehydrogenase, late embryogenesis abundant (LEA) protein, dehydrin LEA-related, dehydrin RAB25) had the highest expression in grains from the plant treated with biofertilizer (Table 2).

**Table 2:** Categories of up-regulated genes function in durum wheat grain based on Gene Ontology (GO) biological process (Panther classification system; REF: triticum aestivum) as affected by biofertilizer consortium under optimal and stress conditions (*n* = 3)

GO biological process complete	Number of differentially expressed genes	Number of expected genes	Fold enrichment	Raw *P*-value	FDR
Response to stress (GO:0006950)	18	6.93	2.6	2.03E-04	4.44E-02
Response to chemical (GO:0042221)	15	4.98	3.01	1.50E-04	3.68E-02
Response to oxygen-containing compound (GO:1901700)	13	1.17	11.08	2.84E-10	5.59E-07
Response to abiotic stimulus (GO:0009628)	10	1.78	5.62	1.44E-05	5.66E-03
Response to inorganic substance (GO:0010035)	10	0.43	23.08	3.66E-11	1.44E-07
Response to temperature stimulus (GO:0009266)	8	0.52	15.45	7.24E-08	9.51E-05
Reproductive process (GO:0022414)	6	0.85	7.09	2.34E-04	4.85E-02
Response to hydrogen peroxide (GO:0042542)	5	0.14	34.92	4.69E-07	2.64E-04
Response to abscisic acid (GO:0009737)	5	0.37	13.58	4.07E-05	1.15E-02
Response to reactive oxygen species (GO:0000302)	5	0.23	21.85	4.35E-06	1.91E-03
Response to alcohol (GO:0097305)	5	0.37	13.58	4.07E-05	1.07E-02
Response to water (GO:0009415)	5	0.1	47.7	1.07E-07	8.41E-05
Response to water deprivation (GO:0009414)	5	0.1	48.89	9.49E-08	9.35E-05
Response to acid chemical (GO:0001101)	5	0.11	46.02	1.27E-07	8.31E-05
Cold acclimation (GO:0009631)	4	0.07	54.9	1.25E-06	6.17E-04
Response to cold (GO:0009409)	4	0.16	24.83	2.54E-05	9.12E-03
Embryo development ending in seed dormancy (GO:0009793)	3	0.06	48.89	4.05E-05	1.33E-02
Embryo development (GO:0009790)	3	0.06	48.89	4.05E-05	1.23E-02

FDR, False Discovery Rate

Test type was Fisher, and A p-value cutoff of 0.05 was considered as a start point.

**Table 3:** Categories of down-regulated genes function in durum wheat grain based on Gene Ontology (GO) biological process (Panther classification system; REF: triticum aestivum) as affected by biofertilizer consortium under optimal and stress conditions (*n* = 3)

GO biological process complete	Number of differentially expressed genes	Number of expected genes	Fold enrichment	Raw *P*-value	FDR
Cellular nitrogen compound metabolic process (GO:0034641)	12	3.15	3.81	2.95E-05	7.28E-03
Gene expression (GO:0010467)	11	1.76	6.24	6.52E-07	2.57E-04
Organic substance biosynthetic process (GO:1901576)	10	2.75	3.63	2.51E-04	4.94E-02
Organonitrogen compound biosynthetic process (GO:1901566)	10	1.3	7.68	3.70E-07	1.62E-04
Cellular macromolecule metabolic process (GO:0044260)	10	2.64	3.79	1.76E-04	3.85E-02
Cellular biosynthetic process (GO:0044249)	10	2.67	3.74	1.96E-04	4.07E-02
Peptide metabolic process (GO:0006518)	9	0.68	13.32	1.60E-08	1.26E-05
Amide biosynthetic process (GO:0043604)	9	0.6	15.01	5.72E-09	5.64E-06
Cellular amide metabolic process (GO:0043603)	9	0.77	11.72	4.73E-08	3.10E-05
Translation (GO:0006412)	9	0.54	16.57	2.45E-09	4.84E-06
Macromolecule biosynthetic process (GO:0009059)	9	1.43	6.29	8.15E-06	2.29E-03
Cellular macromolecule biosynthetic process (GO:0034645)	9	0.91	9.94	1.89E-07	9.32E-05
Cellular nitrogen compound biosynthetic process (GO:0044271)	9	1.44	6.25	8.58E-06	2.25E-03
Peptide biosynthetic process (GO:0043043)	9	0.55	16.32	2.80E-09	3.67E-06
Cellular component biogenesis (GO:0044085)	8	1.01	7.92	5.42E-06	1.78E-03
Ribonucleoprotein complex biogenesis (GO:0022613)	7	0.42	16.85	1.64E-07	9.22E-05
Ribosomal large subunit biogenesis (GO:0042273)	6	0.09	69.8	3.51E-10	1.39E-06
Ribosome biogenesis (GO:0042254)	6	0.34	17.75	1.03E-06	3.69E-04
Translational initiation (GO:0006413)	3	0.08	39.78	6.34E-05	1.47E-02
Maturation of LSU-rRNA (GO:0000470)	3	0.04	82.35	7.54E-06	2.28E-03

FDR, False Discovery Rate

Test type was Fisher, and A p-value cutoff of 0.05 was considered as a start point.

Then, genes involved in other processes such as responding to chemicals (such as nitrogen compound, nutrient, and chemotaxis), and oxygen-containing compounds (such as glucose, lipopolysaccharide, and abscisic acid) were more expressed in grains affected by biofertilizer application, equal to the fold enrichment of 3 and 11.1, respectively (Table 2). Some of these important groups of genes, which are also activated in cellular metabolic process, belonged to the Panther family/subfamily of chitinase 8, xylanase inhibitor protein 1, aldose reductase, glucose/ribitol dehydrogenase, gallate 1-beta-glucosyltransferase, cysteine proteinase inhibitor, sucrose-binding protein, anthocyanidin reductase, UDP-glucosyltransferase, tyrosine-protein phosphatase DSP2, beta-amylase 2, phospholipase A2-beta, an iron-sulfur subunit of complex II, molybdopterin synthase sulphur carrier, cysteine proteinase inhibitor, and bidirectional sugar transporter SWEET15.

### Quantitative assessment of metabolites and stress response systems of plant

**Fig. 5:**
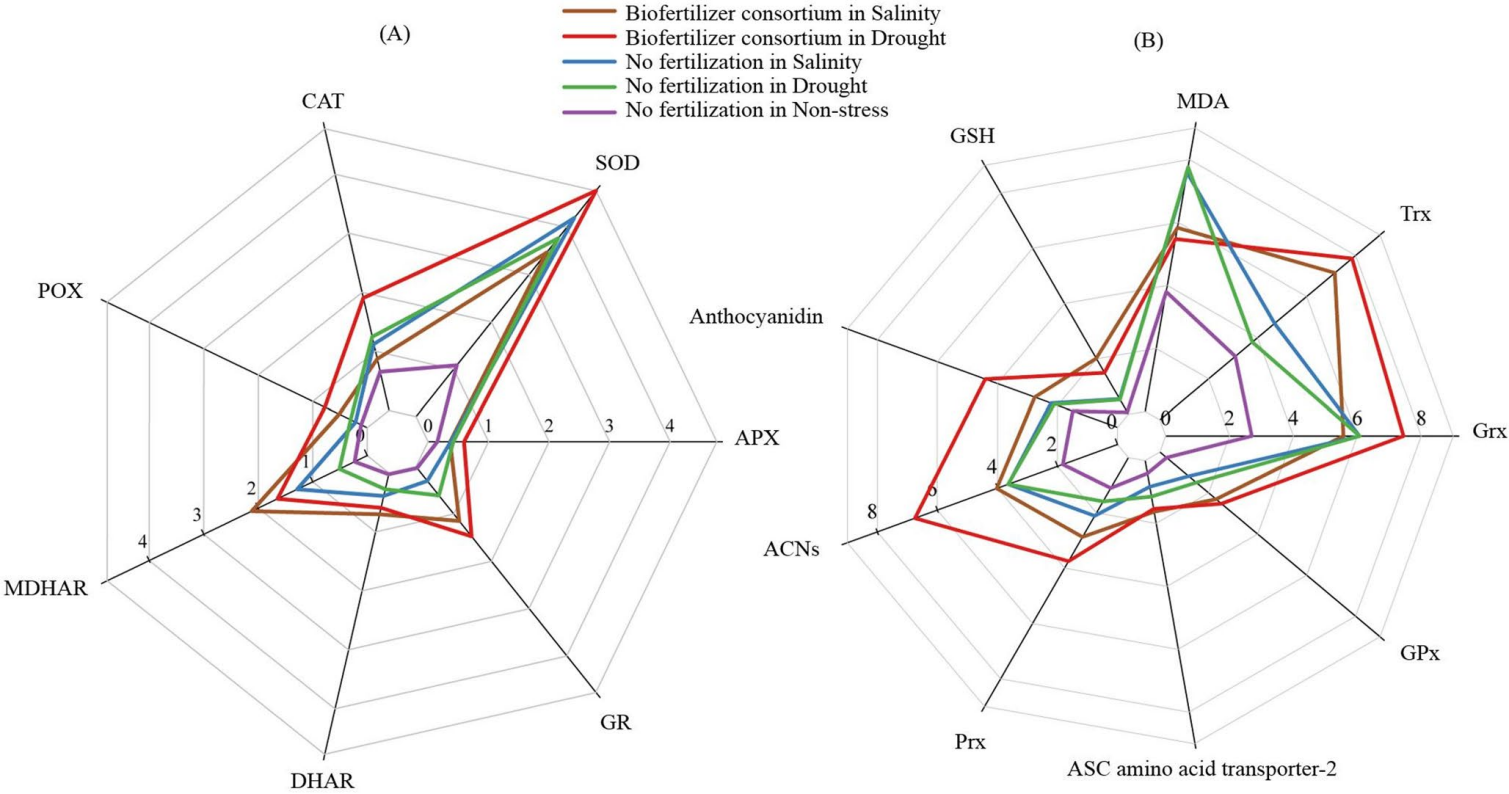
The effect of inoculation with a consortium of four beneficial bacteria and stress treatments on the enzymatic antioxidant (**A**), non-enzymatic components of the antioxidant system and marker of oxidative stress (**B**) antioxidant in wheat grain. The measures within each parameter are presented as the average of 6 replicates (*n* = 6).CAT: Catalase; POX: Peroxidase; SOD: Superoxide dismutase; MDHAR: Monodehydroascorbate reductase; DHAR: Dehydroascorbate reductase; APX: Ascorbate peroxidase; GR: Glutathione reductase; GSH: Glutathione; MDA: Malondialdehyde; Trx: Thioredoxin; Prx: peroxiredoxin; Grx: Glutaredoxin; ACNs: Anthocyanins; GPx: Glutathione peroxidase

A notable finding from the genome-wide transcriptome analysis was the upregulation of 153 differentially expressed genes in response to the treatments, including some that are linked to the antioxidant machinery. To elucidate the significance of this observation, we further investigated the corresponding biochemical parameters related to both the antioxidant and non-antioxidant protective mechanisms in the grains. In this regard, Fig. 5A presents a comparison of treatments in terms of the accumulation of antioxidant enzymes. As confirmed by the comparison of means analysis (Tukey’s HSD test), the concentration of the POX, GR, MDHAR, and DHAR enzymes in the plants inoculated with biofertilizer under stress was significantly (*p* < 0.05) higher than those in no fertilization treatments (Supplementary Table 1). Interestingly, for inoculated plants under salinity, the concentration of CAT, SOD, and APX enzymes was in the same statistical group of unfertilized plants under the same stress, which all were significantly lower than BC treatment in drought (Fig. 5A, Supplementary Table 1A).

All studied non-enzymatic components of antioxidant systems in grains were affected by treatments (Fig. 5B). Accordingly, the maximum concentration of GPx, Prx, Trx, Grx, ACNs, and anthocyanidin was found in BC treatment under drought, which was about 9.6, 3.4, 2.5, 2.4, 3.7, and 3 times higher than Co in non-stress, and 1.9, 1.8, 2.1, 1.1, 1.8, and 2.1 times higher than Co in drought, respectively (Supplementary Table 3B). Whereas BC treatment significantly increased the accumulation of ASC amino acid transporter-2 and GSH in salinity-stressed grains compared to the control in non-stress (+ 4 and + 28.4 times, respectively), this increase was not significant compared to BC in drought-stressed plants, which were 3.8 and 21.4 times higher than control, respectively (Fig. 5B, Supplementary Table 3B). The MDA, as a marker of oxidative stress, respond to biofertilizer differentially from non-enzymatic antioxidants, in which, although its concentration was significantly higher than that in control unstressed plants (+ 44% and + 54%, respectively), it was significantly lower than those unfertilized plants at the same stress level (–42% and − 30%, respectively) (Fig. 5B, Supplementary Table 3B).

**Fig. 6:**
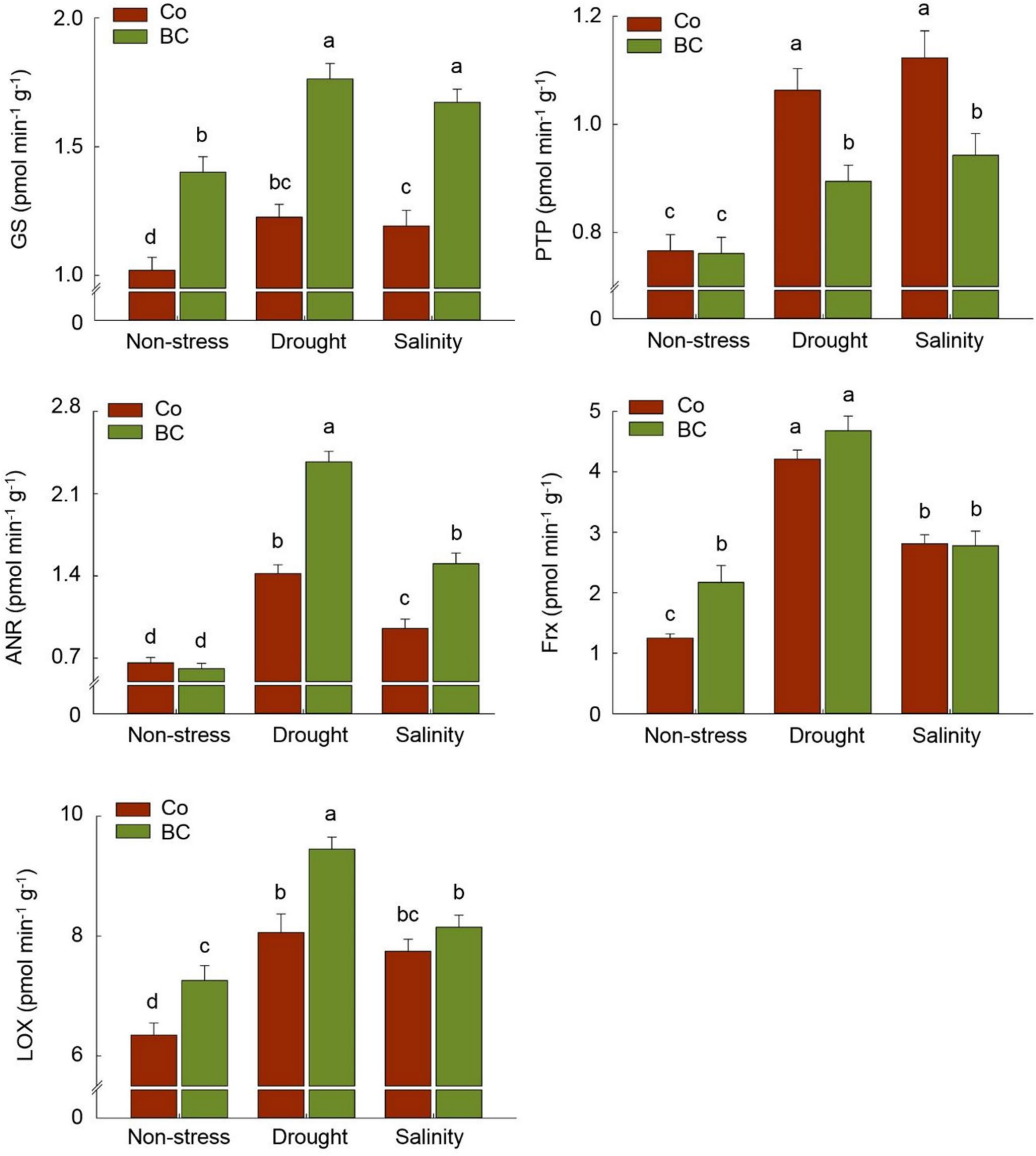
The effect of inoculation with a consortium of four beneficial bacteria and stress treatments on the non-antioxidant enzymes in wheat grain. Means (± standard deviation; *n* = 6) in each parameter followed by similar letter(s) are not significantly different at 5% probability level (Tukey test). GS: Glutamate synthase; PTP: Protein-tyrosine phosphatase; ANR: Anthocyanidin reductase; Frx: Ferredoxin-NADP(H) reductase; LOX: Lipoxygenase

The results of other non-antioxidant enzymes, whose gene expression was changed in response to BC treatment, are set out in Fig. 5. Plant stimulation with BC under stress resulted in a significant increment in the activity of LOX, Frx, and GS in grains, compared to unfertilized plants in non-stress (*p* < 0.05). In contrast, PTP significantly decreased in fertilized plants under both drought (–31%) and salinity (–29%) stresses compared to unfertilized plants (Fig. 6). Moreover, the activity of LOX, GS, and ANR under drought (+ 18%, + 44%, + 91%, respectively) and GS under salinity (+ 40%) in BC-treated plants were significantly higher than those in the same stress levels in unfertilized plants (Fig. 6).

**Fig. 7:**
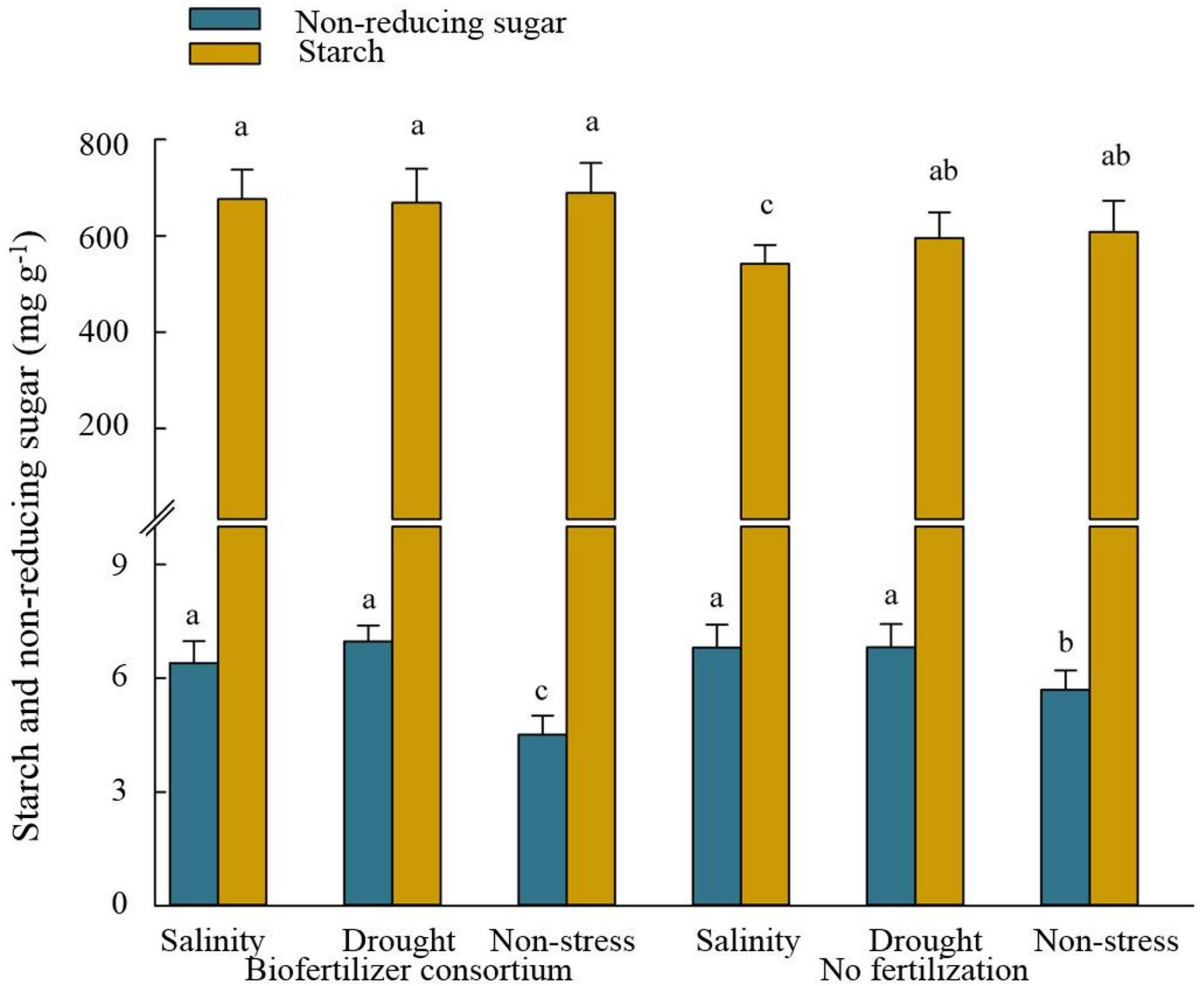
The effect of inoculation with a consortium of four beneficial bacteria and stress treatments on the starch and non-reducing sugar content in wheat grain. Means (± standard deviation; *n* = 6) in each parameter followed by similar letter(s) are not significantly different at 5% probability level (Tukey test)

A better understanding of carbohydrates metabolism and involved enzymes in response to biofertilizers and stress treatments was finally planned. In this regard, although non-reducing sugar content in unfertilized plants was significantly higher under stress than that in non-stress conditions, it was not affected by BC treatment (Fig. 7).

**Fig. 8:**
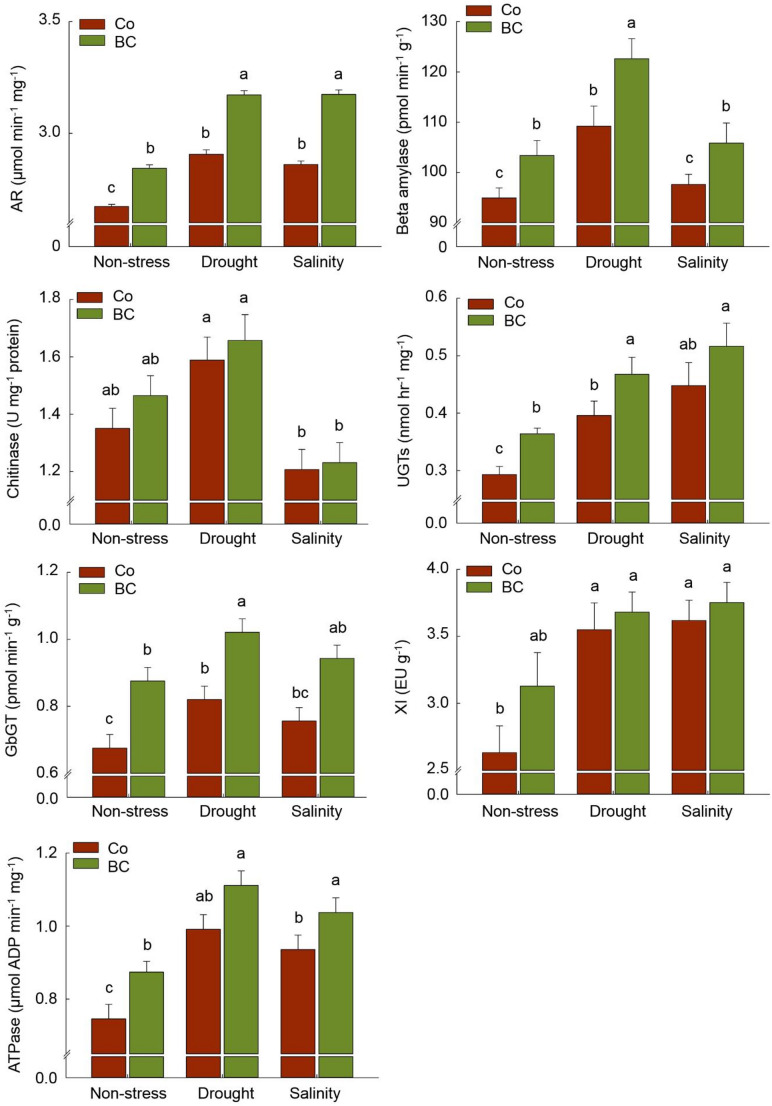
The effect of inoculation with a consortium of four beneficial bacteria and stress treatments on the carbohydrates’ metabolism in wheat grain. Means (± standard deviation; *n* = 6) in each parameter followed by similar letter(s) are not significantly different at 5% probability level (Tukey test). AR: Aldose reductase; GbGT: Gallate 1-beta-glucosyltransferase; XI: Xylanase inhibitor; UGTs: Uridine diphosphate (UDP) glycosyltransferases

Under both stress and non-stress conditions, the increase in beta-amylase (+ 9%-12%), ATPase (+ 11%-17%), UGTs (+ 15%-24%), and GbGT (+ 24%-30%) levels was more pronounced in BC treatment compared to no fertilization. In contrast, aldose reductase (AR) levels in BC-treated plants tended to increase significantly only in stressed plants (+ 10%) compared to unfertilized stressed plants (Fig. 8). Furthermore, the chitinase concentration was affected neither by biofertilization nor by stress treatments. The highest concentration of chitinase in grains was obtained from the drought-stressed plants under both BC and Co fertilization (Fig. 8).

### Germination assessment of harvested grains

**Fig. 9:**
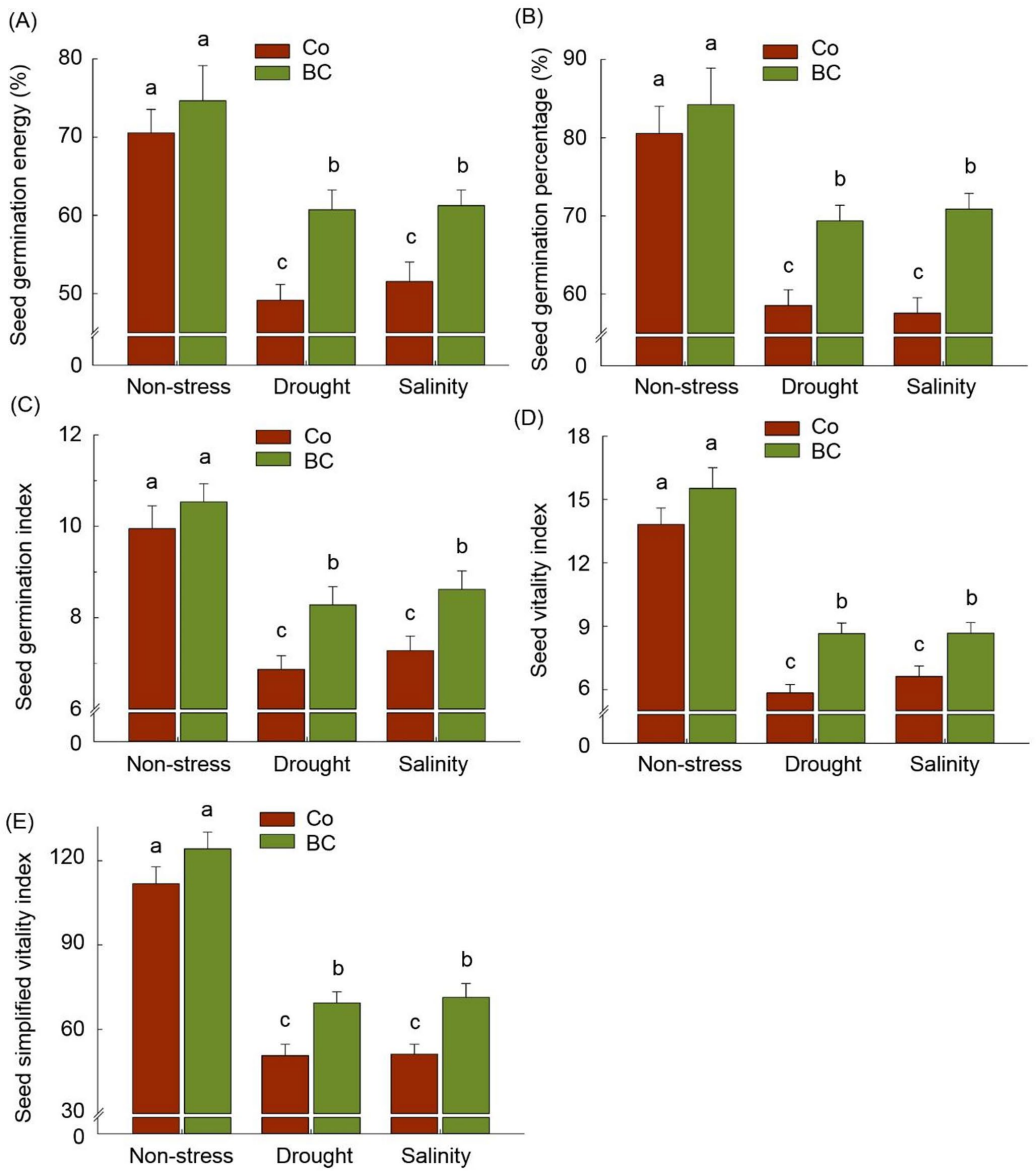
The effect of inoculation with a consortium of four beneficial bacteria and stress treatments on the germination potential of harvested grains. Means (± standard deviation; *n* = 6) in each parameter followed by similar letter(s) are not significantly different at 5% probability level (Tukey test)

The germination potential of harvested grains was assessed to reveal to what extent the biofertilization- and stress-induced changes can affect the grain potential as a seed to establish the next generation of plants. In this regard, the results showed that all germination parameters, including GE, GP, GI, VI, and SVI of grains harvested from stressed plants significantly declined (about − 23–27%) compared to those whose maternal grew up in control conditions. In contrast, however, all germination parameters, including GE, GP, GI, VI, and SVI were not affected by the biofertilization treatment of maternal plants under non-stress conditions (*p* > 0.05), they were clearly improved in the seeds of fertilized plants grown under drought (+ 18%, + 17%, + 21%, + 48%, and + 37%, respectively) and salinity (+ 17%, + 21%, + 19%, + 31%, and + 39%, respectively) stress conditions (*p* < 0.05) (Fig. 9).

## Discussion

Our earlier research highlighted the positive effects of PGPB inoculation on grain yield of unstressed plants (Yaghoubi et al. 2021a). Likewise, under drought and salinity conditions they tended to be at least as effective in lessening the adverse impacts of these stresses on durum wheat plants, as evidenced by physiological and molecular parameters (Yaghoubi et al. 2021c). In order to better understand how these beneficial bacteria act indirectly, this research sought to address some key questions: whether (i) the grain bacterial communities and (ii) transcriptional responses of plant genes changed in response to the bacterial consortium of PGPB strains or stress conditions. In contrast to expectations, neither bacterial community compositions nor diversity indices (Pielou’s Evenness and Shannon entropy) in grains changed significantly compared to the control plants (Supplementary Fig. S1 and Table 1). In contrast to our earlier finding in altering root endophytic bacterial communities in response to PGPB inoculation (Yaghoubi et al. 2021a), no evidence of susceptibility of grain endophytic bacteria was detected to support the previous idea which proposed that the plant microbiome is established by the symbionts and their plant host jointly (Uroz et al. 2019).

Consistent with earlier observations in plant roots (Yaghoubi et al. 2021a), the relative abundances of sequences from the applied bacterial consortium, comprising four beneficial strains from the genera *Acinetobacter* and *Comamonas* for inoculation treatment, were found to be very low or undetectable in the grain endosphere.

Based on both prior experimentation and the current study, it is evident that their survival rate in the soil and re-colonization efficiency inside grains was restricted, or at least our sequencing depth may not have been adequate to accurately capture them. Hence, if we acknowledge that these beneficial bacteria failed to colonize in sufficient quantities to be identified in the grains, potentially explaining their lack of impact on the grain endophytic bacterial community in this study, we cannot disregard previous findings regarding their notable effects on the soil and root bacterial community structure (Yaghoubi et al. 2021a). While the previous findings underscore the necessity for strategies like preservatives to enhance the efficacy and persistence of PGPB inoculants in soil and efficient colonizing inside the plants, the outcomes regarding grain yield, nutrient content, and photosynthetic capacity demonstrated a beneficial impact of the inoculation treatment (Yaghoubi et al. 2021a; 2021b; 2021c; 2022). It appears that the phosphate, potassium, and zinc solubilization, along with nitrogen fixation activity facilitated by PGPB strains during their likely brief survival period, could have positively influenced plants under stress.

One potential reason for the lesser changes observed in grain-associated compared to root endophytic bacteria in our study could be attributed to the absence of plant-mediated mechanisms in the grain in response to the application of the PGPB consortium. These mechanisms may not be as pronounced in the grain as in the root, where the habitat undergoes alterations through adjusted root architecture (Chen et al. 2019; Yaghoubi et al. 2021a). Accordingly, the findings of the current study do not support the previous concept of assembling grain/seed microbiome through horizontal transmission pathways, which could be affected by environmental manipulations (e.g. soil amendments, application of artificial microbial consortia, etc.) (Shade et al. 2017). Therefore, by contrast, there is an immediately dependable conclusion for our case that the bacterial community in grain was shaped solely by plants. This view is somewhat supported by Gundel et al. (2011) who proposed that plant microbiota can be acquired directly from the parent.

Genome-wide analysis was done to identify the plant genes responding to biofertilizer applications and stress treatments. Accordingly, 153 genes were up-regulated (Log_2FC_ ≥ 0.5), and 33 genes were down-regulated (Log_2FC_ ≤ − 0.5) in response to the biofertilizer consortium. A possible explanation for a relatively low number of differentially expressed genes might be related to the type of tissue sample subjected to the analysis, which was mature grain in our study. It was previously reported that a reduction in gene activities occurred in more mature wheat grains compared to early-developing grains (Guan et al. 2022).

Gene ontology enrichment analysis illustrated that most up-regulated sequences were categorized into many stress-related biological processes, which were more pronounced under drought, indicating biofertilizers’ effects in changing stress tolerance in durum wheat plants. Then, to validate the RNA-seq findings and a better comprehension of commonly regulated mechanisms, we further quantitatively measured the products of some expressed genes in the cellular system of grain through biochemical and metabolite analysis.

The higher MDA accumulation in the stressed plants belonged to no fertilization treatment (Fig. 4B), confirming the severe oxidative damage under drought and salinity (Hajihashemi et al. 2021; Angon et al. 2022). This result indicated the stimulation of possible enzymatic and non-enzymatic antioxidant protective mechanisms in PGPB-treated plants to neutralizethe excess ROS generation under stress. Bharti et al. (2016), Singh et al. (2020), and Murali et al. (2021) reported similar findings in PGPR-inoculated plants under salinity and drought, respectively, in particular by producing antioxidant enzymes, ACC deaminase and hormones to cope with stress (Murali et al. 2021; Yaghoubi et al. 2021a). Furthermore, as MDA is regarded to be one of the end products of lipid peroxidation within cells (Zhang et al. 2021), the increase in lipoxygenases (LOX) activity under drought stress (Fig. 5) suggests their role in catalyzing the peroxidation of polyunsaturated fatty acids, ultimately leading to the production of various metabolic regulators under stress conditions (Pokotylo et al. 2015).

Our findings also showed one of the protective mechanisms of fertilized plants upon exposure to drought stress, in which not only the antioxidants’ direct scavenging enzymes (CAT, POX, and SOD) but also the expression of related genes (e.g. catalase isozymes, catalase 3 (Cat3-A1), peroxidase 55-like and peroxygenase-like) were significantly enhanced as compared to no fertilization treatments, a mechanism that was not activated in fertilized plants under salinity stress (Fig. 4B). The observation under drought in this study mirrors those of our previous study which suggested that the application of a biofertilizer consortium could indirectly help maintain the activity of enzymes involved in catalyzing the oxidation-reduction reactions by stimulating the accumulation of polyphenols and ferric-reducing antioxidant power (Yaghoubi et al. 2022), as those metabolites can form complexes with metals that catalyze oxygenation reactions (Yaghoubi et al. 2022; Notununu et al. 2022). In contrast, the somehow contradictory findings under salinity may be due to the reaching of a steady-state point or the exhausting of the antioxidant enzymes system under severe salinity conditions (Lu et al. 2021; Angon et al. 2022), which led to a sharp decrease in the efficiency of this system in regulating ROS detoxification.

The elevated levels of GSH and the up-regulation of associated genes such as glutathione S-transferase under salinity, as opposed to drought, in PGPB-treated plants suggest the potential activation of alternative detoxification pathways in stressed plants to mitigate oxidative damage. Accordingly, consistent with up-regulated genes that are responsible for encoding the key enzymes in the ascorbate-glutathione (ASC/GSH) cycle, there seems to be some evidence of higher activation of some antioxidant components involved in the ASC/GSH pathway (GSH, GPX, GR, APX, and MDHAR) in response to biofertilizer under salinity and/or drought stress conditions (Fig. 4A, B). Similarly, increased expression of some antioxidant enzymes genes (e.g. GR, MDHAR, and APX) and their activity in stressed-plant tissues was previously reported in PGPB-treated plants (Bharti et al. 2016; Li et al. 2020). In this regard, increased activation of MDHAR in PGPB-treated plants under stress corroborates earlier findings by Timmusk et al. (2014) and Erice et al. (2017), reporting that this ROS-scavenging key enzyme converts monodehydroascorbate to reduce ascorbate in the ASC/GSH cycle in inoculated plants (Noctor and Foyer 1998). Erice et al. (2017) found that the increase in MDHAR coincided with an increase in ATPase activity, the enzyme involved in the regulation of energy status in plant tissues, which contributed to proton pumping, sodium compartmentalization regulating, and osmotic adjusting, leading to an increase in antioxidant protective system (Chen et al. 2015). In this regard, as shown in Fig. 6, ATPase activity in stressed plants, in particular in those treated by PGPB, was significantly higher than in control plants, which was consistent with higher expression of related genes including V-type proton ATPase and Plasma membrane ATPase 1-like in our research. Moreover, the increment of MDHAR and GSH accumulation led to an increase in the concentration of DHAR enzyme in the present study (Fig. 4A), as a member of the omega class glutathione-S-transferases since DHAR uses GSH to reduce the monodehydroascorbate produced from the activity of MDHAR to dehydroascorbate in the ASC/GSH cycle (Hao et al. 2019).

Wells and Xu (1994) proposed the non-enzymatic reduction of dehydroascorbate, suggesting the activation of proteins like glutaredoxins (Grx). Our study observed this phenomenon in fertilized plants under drought stress (Fig. 4B). RNA sequencing revealed increased expression of homocysteine S-methyltransferase 4 and decreased expression of cysteine proteinase inhibitor 4, cysteine proteinase inhibitor TrcC-6, cysteine proteinase inhibitor 8, and cysteine protease RD19D under drought (Supplementary Table 2). This altered expression pattern of genes encoding enzymes involved in cysteine biosynthesis and inhibition suggests a link between cysteine function, redox signaling, and the accumulation of protective proteins like Trx (Mata-Pérez and Spoel 2018). As can be seen from Fig. 4B, increased Trx molecule content in our study resulted in higher activity of Prx and GPx in PGPB-treated plants, probably through supplying the reducing power to reductases, which contribute to the lipid hydroperoxides degradation or oxidized proteins reparation (Jedelská et al. 2020). Furthermore, higher Trx content in drought-stressed plants could be one of the reasons for the increased activity of the antioxidant direct scavenging enzymes (CAT, POX, and SOD) under drought, compared to the salinity in fertilized plants, since Trx is known as a protective agent for preserving antioxidant enzymes from inhibition factors or severe oxidative damage (Mata-Pérez and Spoel 2019).

Genes involved in sugar metabolism are among the most up-regulating groups in the grain of fertilized plants. Transcriptome pattern of NADH-dependent glutamate synthase 1gene (Supplementary Table 2) and GS activity (Fig. 5) in PGPB-treated plants seem to be consistent with other research reporting higher accumulation of GS enzyme under stress which can utilize glutamate as a precursor, and regulate proline and non-reducing sugars (e.g., sucrose) synthesis pathway (Yin et al. 2022). The increment of glutamate and proline content was already observed in fertilized plants in our previous study within the same experimental setup (Yaghoubi et al. 2022). Also, quantitative estimation of AR (Fig. 7) confirmed the results of overexpression of related genes in grain. Although, some previous research reported that AR accumulation could result in oxidative stress through increasing sorbitol accumulation and competing with GR for NADPH (Ramana 2011), it’s important to interpret these findings cautiously. Our results show that GR activity increased significantly in fertilized plants under stress conditions (Fig. 4A), indicating that this competition may not substantially impact. On the other hand, some further research suggested that AR accumulation in mature grains and vegetative tissues has been associated with stress tolerance via switching on the redox-sensitive transcription factors and detoxification of biogenic and xenobiotic aldehydes to their corresponding alcohols during oxidative stress (Li and Foley 1995; Sree et al. 2000; Ramana 2011). Another set of crucial enzymes involved in detoxification under stress are glycosyltransferases, such as UGTs and GbGT (Ono et al. 2016; Ahn et al. 2021; Campos et al. 2022), which were notably up-regulated under biofertilization treatment. Although, Contrary to UGTs, the activity of the GbGT enzyme in the grain exhibited a non-significant increase when compared to the unfertilized plants under the same stress level (Fig. 7). UGTs, by catalyzing the glycosyl-transferring reaction, are involved in synthesizing the oligosaccharides through transferring the activated monosaccharide donors to carbohydrates, and producing the various secondary metabolites, especially anthocyanins (Ahn et al. 2021; Grauso et al. 2023). Hence, a potential connection could exist between the overexpression of UGTs and GbGT genes and the accumulation of non-reducing sugars and anthocyanins in plants, as depicted in Figs. 4B and 6, respectively. The biosynthesis of tannin is also reported as one of the key metabolites catalyzed by glycosyltransferase enzymes (Ono et al. 2016). Although the present research did not focus on the tannin content, the higher content of anthocyanidins and ANR in the plants under drought (Figs. 4B and 5) provided a piece of evidence in biosynthesizing such flavonoid compounds in plants, especially in response to biofertilizer treatment. Therefore, there is a possible relationship between glycosyltransferase enzymes and anthocyanidins content, since the ANR enzyme is active in converting anthocyanidins into the corresponding flavonoid (Xie et al. 2004).

In line with our observations, Lv et al. (2022) previously reported an increase in the expression of genes responsible for chitinase in plants under drought stress conditions, despite the absence of chitin in the plant. However, our findings did not consistently align with the effect of biofertilizer on the overexpression of these genes when estimating the concentration of the chitinase enzyme (Fig. 7). Nonetheless, we observed a significant increase in the concentration of beta-amylase under both drought and salinity stress conditions. Interestingly, this increase in response to biofertilizer in drought-stressed plants was notably higher than in salinity-stressed plants (Fig. 7), consistent with the pattern of expression of the corresponding gene, Beta-amylase 2 (Supplementary Table 2). This finding, together with the overexpression of endogenous alpha-amylase/subtilisin inhibitor in our study, can somehow justify the no increase of starch content in fertilized plants under drought compared to salinity and non-stress conditions (Fig. 6), since higher beta-amylase accumulation is usually associated with higher starch breakdown in plants (Reinhold et al. 2011).

The expression of tyrosine-protein phosphatase DSP2 was detected to be manifold decreased in biofertilizer-treated plants under stress, consistent with the reduction of PTP enzyme activity in the same treatment as compared to the unfertilized plants (Fig. 5). The reason for this is not clear but it may have something to do with the biosynthesis of abscisic acid (ABA) as one of the main functions of this family of genes (Shankar et al. 2015). Similarly, Berrocal-Lobo et al. (2011) suggested that the DSP protein is linked to increased sensitivity of plants under stress.

**Fig. 10:**
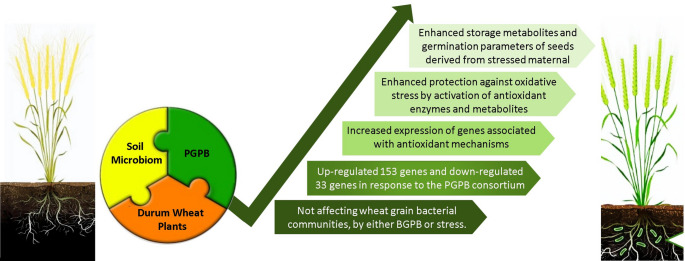
A comprehensive overview of the mechanisms induced by PGBP, summarizing our research findings

The germination potential of the progeny of unfertilized stressed maternal plants significantly declined compared to those of control plants. While we cannot exclude the possibility of genetic and epigenetic (maternal) effects in the present research, we can at least confirm that exposure to stress in one generation adversely affects the subsequent germination of seeds if not exposed to the same stress. Moreover, all studied germination parameters of seeds were affected by the biofertilization treatment of maternal plants. It may be associated with changes in the reservoir of seed storage metabolites, as it was shown in the present and previous research (Yaghoubi et al. 2022), that the seeds and seedlings rely on during early growth (Hatzig et al. 2018). This finding can somehow confirm that however the germination potential of seeds of the next generation is not affected by horizontal transfer from the surrounding habitat of stressed maternal plants; it can be acquired by vertical gene transfer from the PGPR-treated maternal. Nevertheless, caution must be applied, as the findings do not match those observed in earlier studies regarding the role of microbial elements of the maternal plants in vertical gene transfer to the next generation (Mitter et al. 2017; Arif et al. 2020) since no changes were observed in seed microbiome in the present research. It can be also considered that the adaptation strategy of progeny may exhibit bias independent of DNA sequence alterations, known as an epigenetic effect. This involves the formation of transcriptional memory and inheritable changes in the phenotype of PGPR-treated plants (Tiwari et al. 2022; Labella et al. 2024). In this regard, Hatzig et al. (2018) suggested that the vigor of progeny seeds and seedlings could be improved mainly because of inter-generational memory. Hence, it can be concluded that the interactions between PGPR and maternal plants under stress conditions are crucial for influencing the seed germination process, leading to a higher level of uniform germination rate in progeny. Moreover, our previous research (Yaghoubi et al. 2022) demonstrated that inoculated plants showed 78% and 73% increases in grain production under drought and salinity stress conditions, respectively, compared to unfertilized controls. The observed improvements in germination parameters in the inoculated plants suggest that enhanced germination potential in biofertilized seeds under stress may directly contribute to higher yield. Therefore, these findings highlight the potential for biofertilization to not only improve germination parameters but also increase overall grain production. A comprehensive overview of the mechanisms induced by PGBP is summarized in Fig. 10, which outlines our research.

## Conclusion

The current study aimed to contribute significantly to fill the gaps in understanding how plant grains establish and sustain their microbiome and alter gene expression patterns in response to bacterial consortium fertilization. The primary finding was that neither biofertilizers nor stress treatments had significant effects on the bacterial communities and diversity within wheat grain. However, transcriptomic analyses and metabolite assessments revealed significant alterations in molecular mechanisms within durum wheat plants treated with PGPB. Moreover, the interactions between PGPR and maternal plants under stress conditions are critical to affect the seed germination process and provide a higher level of homogeneous germination rate in progeny. Accordingly, the present research highlighted the potential uses of beneficial bacteria in inducing stress tolerance in plants, especially under drought, through shifting transcriptome expression of genes involved in antioxidant and non-antioxidant protective systems along with sugar metabolism.

## Supplementary Information

Below is the link to the electronic supplementary material.

Supplementary Material 1

## Data Availability

The data that support the findings of this study are available from the corresponding author upon reasonable request. Also, a total of 57.115 gigabytes of experimental data was uploaded to Sequence Read Archive (SRA) with the accession number SRP105363.

## Abbreviations

ACNs: Anthocyanins
ANR: Anthocyanidin reductase
AR: Aldose reductase
APX: Ascorbate peroxidase
BC: Biofertilizer consortium of four PGPB strains
CAT: Catalase
Co: No fertilization (control)
DHAR: Dehydroascorbate reductase
Frx: Ferredoxin-NADP(H) reductase
GbGT: Gallate 1-beta-glucosyltransferase
GPx: Glutathione peroxidase
GR: Glutathione reductase
Grx: Glutaredoxin
GS: Glutamate synthase
GSH: Glutathione
LOX: Lipoxygenase
MDA: Malondialdehyde
MDHAR: Monodehydroascorbate reductase
PGPB: Plant growth-promoting bacteria
POX: Peroxidase
Prx: peroxiredoxin
PTP: Protein-tyrosine phosphatase
SOD: Superoxide dismutase
Trx: Thioredoxin
UGTs: Uridine diphosphate (UDP) glycosyltransferases
XI: Xylanase inhibitor
