# Supplementary material for: Transcriptomic, biochemical, and microbiome assessments into drought and salinity tolerance in durum wheat mediated by plant growth-promoting bacteria
Source: Physiol Mol Biol Plants. 2025 Nov 19;31(12):2121–43. doi: 10.1007/s12298-025-01686-z (PMC12715099; doi:10.1007/s12298-025-01686-z)
Supplement: Supplementary file 1 — Supplementary Material 1 [file 12298_2025_1686_MOESM1_ESM.docx]

| **Supplementary Table S1.** The relative abundance (%) of genera *Acinetobacter* and *Comamonas* in the grain samples as affected by biofertilizer consortium under optimal and stress conditions (n = 3). | | | | | | | |
| --- | --- | --- | --- | --- | --- | --- | --- |
|  | Fertilization | |  | | Stress | | |
| Genera | C | I | |  | Control | Drought | Salinity |
| *Comamonas* | 0.000 | 0.003 | |  | 0.001 | 0.003 | 0.002 |
| *Acinetobacter* | 0.002 | 0.007 | |  | 0.003 | 0.006 | 0.003 |

| **Supplementary Table S2.** The detected genes in wheat grains with the differentially expressed pattern as affected by biofertilizer consortium under optimal and stress conditions. Bold values represent the statistically significant differences in gene expression (False Discovery Rate (FDR) < 0.05). (n = 3)  DS: Drought stress; SS: Salinity stress; NS: Non-stress: Co: No fertilization (control); BC: Biofertilizer consortium of four PGPB strains. | | | | | | | | |
| --- | --- | --- | --- | --- | --- | --- | --- | --- |
| Genes | NS | DS | SS | Co | Co | BC | BC |  |
|  | BC vs Co | BC vs Co | BC vs Co | DS vs NS | SS vs NS | DS vs NS | SS vs NS |  |
| Translocator protein homolog (LOC123100358), mRNA | **9.5E-05** | 4.7E-01 | 1.4E-01 | 1.0E+00 | **3.9E-02** | **1.6E-03** | **3.2E-02** |  |
| Oleosin 18 kDa-like (LOC123094303), mRNA | **4.1E-04** | **2.1E-04** | 1.9E-01 | 1.0E+00 | **1.8E-02** | **8.4E-04** | 5.1E-01 |  |
| Cupincin-like (LOC123105370), mRNA | **2.1E-10** | **2.1E-08** | 4.8E-01 | 1.9E-01 | 5.6E-01 | **4.2E-03** | **9.1E-10** |  |
| Probable inactive leucine-rich repeat receptor kinase XIAO | **4.8E-03** | 9.8E-01 | **3.6E-02** | 1.0E+00 | **6.4E-14** | **3.4E-05** | 1.0E+00 |  |
| Sucrose-binding protein (LOC104582620), mRNA | 8.3E-01 | **2.2E-02** | 4.5E-01 | 1.0E+00 | **3.5E-02** | 9.7E-01 | 1.0E+00 |  |
| Cysteine proteinase inhibitor TrcC-6 mRNA | 2.1E-01 | 7.2E-01 | **3.7E-02** | 1.0E+00 | 5.9E-01 | **2.3E-12** | **6.6E-03** |  |
| Wheatwin-2-like (LOC123072755), mRNA | 9.9E-01 | **7.7E-06** | 6.2E-01 | 1.9E-01 | 9.0E-01 | 5.1E-02 | **7.8E-03** |  |
| Uncharacterized LOC123133789 (LOC123133789), mRNA | 3.9E-01 | **2.0E-04** | 4.5E-01 | 1.0E+00 | **8.2E-03** | 7.3E-02 | 1.0E+00 |  |
| 28S ribosomal RNA (LOC123074074), rRNA | **1.1E-02** | 4.6E-01 | 9.3E-01 | 1.0E+00 | 8.4E-01 | 8.0E-02 | **2.0E-05** |  |
| Em protein-like (LOC123052768), mRNA | **4.0E-03** | 6.4E-01 | 1.0E+00 | 1.0E+00 | 9.5E-01 | **1.4E-03** | **1.4E-02** |  |
| Uncharacterized LOC123100673 (LOC123100673), mRNA | **5.8E-03** | 9.2E-02 | 2.9E-01 | 1.0E+00 | 5.1E-01 | **2.8E-02** | **1.6E-02** |  |
| Histone H1-like (LOC119356006), mRNA | **2.0E-03** | 9.7E-01 | 4.8E-01 | 8.3E-02 | 4.1E-01 | **2.6E-04** | **7.4E-03** |  |
| Organic cation/carnitine transporter 4-like (LOC123190581), mRNA | **1.1E-02** | 8.0E-01 | 6.2E-01 | 1.0E+00 | 6.7E-01 | **1.6E-02** | **6.6E-03** |  |
| Late embryogenesis abundant protein 18-like (LOC123042020), mRNA | **5.0E-07** | 9.2E-02 | 1.0E+00 | 1.0E+00 | **1.1E-02** | 1.8E-01 | 1.0E+00 |  |
| Glycerophosphodiester phosphodiesterase GDPD2-like (LOC123062398), mRNA | **6.4E-09** | 3.1E-01 | 9.7E-01 | 1.0E+00 | 8.2E-01 | **2.4E-06** | **4.2E-03** |  |
| Lysosomal amino acid transporter 1 homolog (LOC123069643), mRNA | **1.3E-03** | NA | NA | 1.0E+00 | NA | **1.2E-02** | **3.1E-02** |  |
| Glycine-rich RNA-binding protein blt801 (LOC123087309), mRNA | **8.9E-04** | 7.9E-01 | 4.8E-01 | 1.0E+00 | **9.8E-03** | **1.8E-02** | 9.6E-01 |  |
| Glycine-rich RNA-binding protein blt801-like (LOC119294603), mRNA | **7.1E-04** | 7.8E-01 | 1.7E-01 | 1.0E+00 | **8.2E-03** | 6.1E-01 | **5.4E-03** |  |
| Late embryogenesis abundant protein D-34-like (LOC123116966), mRNA | **5.1E-05** | 1.5E-01 | 6.1E-01 | 1.0E+00 | 5.6E-01 | **2.4E-03** | **4.1E-03** |  |
| Probable calcium-binding protein CML15 (LOC123142464), mRNA | 9.4E-01 | 9.5E-01 | 2.0E-01 | 1.0E+00 | NA | **3.6E-02** | **8.6E-03** |  |
| 1-Cys peroxiredoxin PER1 (LOC119367460), mRNA | 9.8E-01 | 5.6E-02 | 3.6E-01 | 1.0E+00 | **4.5E-02** | **3.3E-02** | **3.5E-03** |  |
| Protein translation factor SUI1 homolog (LOC543434), mRNA | 9.7E-01 | 5.2E-01 | 5.0E-01 | 1.0E+00 | **3.7E-02** | **4.6E-02** | 1.0E+00 |  |
| Probable calcium-binding protein CML16 (LOC119266762), mRNA | 4.6E-01 | NA | NA | 1.0E+00 | NA | 4.3E-01 | **1.4E-02** |  |
| 17.5 kDa class II heat shock protein (LOC119267259), mRNA | 8.4E-01 | NA | NA | 1.0E+00 | NA | **5.5E-04** | **8.6E-03** |  |
| NADH-dependent glutamate synthase 1 mRNA, partial cds | NA | **2.3E-10** | NA | 1.0E+00 | NA | **5.9E-12** | **8.7E-04** |  |
| Glutathione S-transferase 1 (LOC123069127), mRNA | 9.1E-01 | 1.4E-01 | **8.2E-03** | 1.0E+00 | 6.2E-01 | 2.7E-01 | **2.2E-04** |  |
| 1,2-dihydroxy-3-keto-5-methylthiopentene dioxygenase 2 (LOC100415845), transcript variant X2, mRNA | 5.3E-01 | 4.6E-01 | 6.2E-01 | 1.0E+00 | **3.9E-02** | 2.3E-01 | 1.0E+00 |  |
| Glutathione S-transferase 1-like (LOC123090955), mRNA | 2.1E-01 | 6.2E-01 | **9.8E-04** | 1.0E+00 | **3.7E-02** | 3.4E-01 | **5.6E-03** |  |
| Uncharacterized LOC123089428 (LOC123089428), mRNA | 4.9E-01 | 4.8E-01 | 9.7E-01 | 1.0E+00 | **3.9E-02** | **2.7E-02** | 1.0E+00 |  |
| Late embryogenesis abundant protein 3-like (LOC123106376), mRNA | 6.8E-02 | 4.7E-01 | 1.1E-01 | 1.0E+00 | **1.2E-03** | **1.8E-04** | 1.0E+00 |  |
| Malate dehydrogenase, glyoxysomal-like (LOC123105182), mRNA | 8.2E-01 | 9.5E-01 | 6.0E-01 | 1.0E+00 | **4.7E-02** | 6.7E-01 | 1.0E+00 |  |
| Receptor-like protein kinase 7 (LOC123110112), mRNA | 9.9E-01 | 9.5E-01 | 9.0E-01 | 1.0E+00 | **8.2E-03** | 8.5E-02 | 1.0E+00 |  |
| Late embryogenesis abundant protein D-34-like (LOC123115467), mRNA | 1.8E-01 | 9.9E-01 | 8.2E-01 | 1.0E+00 | **8.4E-07** | 6.1E-02 | 1.0E+00 |  |
| Catalase isozyme 2-like (LOC119319548), mRNA | 3.4E-01 | **3.4E-02** | 9.7E-01 | 1.0E+00 | 7.6E-02 | **2.9E-03** | 7.4E-02 |  |
| DIMBOA UDP-glucosyltransferase BX8-like (LOC123130157), mRNA | 8.9E-02 | 8.0E-01 | 6.2E-01 | 1.0E+00 | **2.0E-02** | 4.4E-01 | 1.0E+00 |  |
| Protein KINESIN LIGHT CHAIN-RELATED 1-like (LOC123135501), mRNA | NA | NA | NA | 1.0E+00 | NA | **3.0E-05** | **1.6E-02** |  |
| 26 kDa endochitinase 2 (LOC119327420), mRNA | 9.9E-01 | 2.5E-01 | 9.4E-01 | 1.0E+00 | 9.9E-01 | **9.6E-03** | **2.5E-02** |  |
| Glucose and ribitol dehydrogenase homolog (LOC123087893), mRNA | 9.9E-01 | **9.7E-03** | **7.4E-04** | 1.0E+00 | 7.0E-01 | **2.5E-02** | 1.0E+00 |  |
| Retrotransposon Angela, partial sequence; retrotransposon Wilma, complete sequence; and ZCCT2-B2a (VRN2) gene, complete cds | 7.5E-01 | **7.0E-10** | **1.8E-02** | 1.0E+00 | 7.5E-01 | **4.1E-02** | 1.0E+00 |  |
| Gamma gliadin-B1, gamma gliadin-B2, delta gliadin-B1, gamma gliadin-B4, gamma gliadin-B6, omega gliadin-B3, omega gliadin-B6, LMW-B2, and LMW-B3 genes, complete cds | 9.6E-01 | **2.0E-02** | **3.5E-02** | 1.0E+00 | 9.0E-01 | **3.5E-03** | 8.2E-01 |  |
| Late embryogenesis abundant protein D-34-like (LOC119293900), mRNA | 9.3E-01 | **1.1E-02** | **3.6E-03** | 1.0E+00 | 3.0E-01 | 1.7E-01 | 9.6E-01 |  |
| Embryonic protein DC-8-like (LOC119331090), mRNA | **3.7E-02** | **1.0E-07** | 6.2E-01 | 1.0E+00 | 3.5E-01 | 5.7E-02 | 2.2E-01 |  |
| 28S ribosomal RNA (LOC123171826), rRNA | **1.1E-11** | **5.9E-08** | 1.0E+00 | 1.0E+00 | 4.1E-01 | 1.2E-01 | 1.0E+00 |  |
| Vicilin-like seed storage protein At2g28490 (LOC123092206), mRNA | **4.7E-02** | **3.5E-03** | 8.4E-01 | 1.0E+00 | 5.5E-01 | **2.3E-02** | **2.9E-02** |  |
| 28S ribosomal RNA (LOC123074282), rRNA | 9.8E-01 | 6.4E-01 | **7.8E-05** | 1.0E+00 | 3.5E-01 | **1.2E-02** | 3.2E-01 |  |
| 28S ribosomal RNA (LOC123074164), rRNA | 9.9E-01 | 9.8E-01 | **5.6E-22** | 1.0E+00 | 9.3E-01 | 3.4E-01 | 7.8E-01 |  |
| Glutelin type-B 1-like (LOC123189207), misc_RNA | 8.1E-01 | **1.1E-02** | 9.9E-01 | 1.0E+00 | NA | 6.8E-01 | 1.0E+00 |  |
| Probable glutathione S-transferase GSTF1 (LOC123190330), mRNA | 9.9E-01 | 9.0E-01 | **2.5E-03** | 1.0E+00 | 3.0E-01 | 5.9E-01 | **3.6E-02** |  |
| Ribosome biogenesis protein NOP53-like (LOC123190882), mRNA | 9.6E-01 | **1.9E-02** | 7.4E-01 | 1.0E+00 | 9.3E-01 | 4.7E-01 | 1.0E+00 |  |
| Aquaporin TIP3-1 (LOC123120564), mRNA | 9.9E-01 | **5.0E-05** | 9.9E-01 | 1.0E+00 | 9.9E-01 | 2.5E-01 | 1.0E+00 |  |
| Secretory protein (WAS-2) mRNA, complete cds | 1.0E+00 | **4.7E-02** | 6.2E-01 | 1.0E+00 | 9.9E-01 | **1.4E-03** | 9.6E-01 |  |
| EC protein III-like (LOC543366), mRNA | 8.5E-01 | **5.4E-03** | 6.7E-01 | 1.0E+00 | 8.7E-01 | 1.6E-01 | 1.0E+00 |  |
| Late embryogenesis abundant protein 19-like (LOC123054223), mRNA | 2.2E-01 | **3.7E-05** | 9.6E-01 | 1.0E+00 | 9.3E-01 | **4.5E-02** | 3.6E-01 |  |
| Uncharacterized LOC123056734 (LOC123056734), mRNA | 9.9E-01 | **1.3E-02** | 9.3E-01 | 1.0E+00 | 3.5E-01 | 1.0E-01 | 5.8E-01 |  |
| Bowman-Birk type wound-induced protease inhibitor (tsbbi) gene, complete cds | 9.1E-01 | **1.1E-02** | 9.8E-01 | 1.0E+00 | 9.9E-01 | **9.5E-04** | 1.7E-01 |  |
| UPF0098 protein CPn_0877/CP_0992/CPj0877/CpB0906-like (LOC123059798), mRNA | 9.9E-01 | **3.9E-05** | 1.0E+00 | 1.0E+00 | 9.9E-01 | **2.2E-03** | 1.0E+00 |  |
| Aldose reductase (LOC119308177), mRNA | **9.9E-03** | **2.0E-02** | NA | 1.0E+00 | NA | **6.3E-04** | **1.0E-02** |  |
| Late embryogenesis abundant protein, group 3-like (LOC543476), transcript variant X1, mRNA | 9.9E-01 | **3.0E-06** | 6.3E-01 | 1.0E+00 | 9.0E-01 | **1.8E-05** | 9.6E-01 |  |
| Uncharacterized LOC123182779 (LOC123182779), mRNA | 9.7E-02 | 1.7E-01 | **2.2E-03** | 1.0E+00 | 1.5E-01 | 5.6E-01 | 1.6E-01 |  |
| Xylanase inhibitor protein 1-like (LOC119301263), mRNA | 6.5E-01 | **1.6E-08** | 9.0E-01 | **4.3E-02** | **7.4E-03** | **2.5E-02** | 1.0E+00 |  |
| TIP3;1 mRNA for tonoplast intrinsic protein 3;1, complete cds | 9.9E-01 | **1.3E-02** | 9.4E-01 | 1.0E+00 | 9.4E-01 | 8.0E-02 | 1.0E+00 |  |
| Uncharacterized LOC123122480 (LOC123122480), mRNA | 9.9E-01 | **3.7E-05** | 9.7E-01 | 1.0E+00 | 8.0E-01 | 1.8E-01 | 1.0E+00 |  |
| EC protein I/II-like (LOC543479), mRNA | 9.7E-02 | **5.3E-03** | 9.0E-01 | 1.0E+00 | 8.4E-01 | 8.2E-01 | 1.0E+00 |  |
| Triticum aestivum uncharacterized LOC123128156 (LOC123128156), mRNA | 9.9E-01 | **4.6E-02** | 9.6E-01 | 1.0E+00 | 9.8E-01 | 4.0E-01 | 1.0E+00 |  |
| UPF0098 protein CPn_0877/CP_0992/CPj0877/CpB0906-like (LOC119348552), mRNA | 9.9E-01 | **8.3E-03** | 9.5E-01 | 1.0E+00 | 9.3E-01 | **1.9E-02** | 1.0E+00 |  |
| Aldose reductase (LOC123136851), mRNA | 9.9E-01 | **1.2E-09** | **6.9E-03** | 1.0E+00 | 9.2E-01 | **1.2E-02** | **1.0E-02** |  |
| ABA-inducible protein PHV A1 (LOC542804), transcript variant X3, mRNA | 9.9E-01 | **1.6E-02** | 9.7E-01 | 1.0E+00 | NA | **6.7E-04** | 1.0E+00 |  |
| ATP-dependent RNA helicase SUV3, mitochondrial-like (LOC119303115), mRNA | 9.9E-01 | **2.4E-02** | 9.7E-01 | 1.0E+00 | 9.8E-01 | 2.5E-01 | 1.0E+00 |  |
| UDP-glucosyltransferase 29-like (LOC123184767), mRNA | 9.9E-01 | **4.5E-02** | **4.7E-02** | **3.6E-02** | 7.4E-01 | **4.6E-02** | **4.0E-02** |  |
| 60S ribosomal protein L35-2-like (LOC123189008), mRNA | 9.9E-01 | **3.0E-03** | 9.9E-01 | 1.0E+00 | 9.8E-01 | **2.7E-04** | 1.0E+00 |  |
| Eukaryotic translation initiation factor 3 subunit H-like (LOC123189015), transcript variant X2, mRNA | 9.9E-01 | **5.4E-03** | 9.7E-01 | 1.0E+00 | 9.8E-01 | 7.4E-02 | 1.0E+00 |  |
| 11-beta-hydroxysteroid dehydrogenase 1A-like (LOC119354979), mRNA | 9.4E-01 | **1.6E-02** | **8.4E-03** | 1.0E+00 | 7.5E-01 | **1.6E-02** | 1.0E+00 |  |
| Uncharacterized LOC123185498 (LOC123185498), mRNA | 8.4E-01 | **1.6E-03** | 6.2E-01 | 1.0E+00 | 2.2E-01 | 6.0E-01 | 1.0E+00 |  |
| Endogenous alpha-amylase/subtilisin inhibitor-like (LOC119359713), mRNA | 9.9E-01 | **1.5E-02** | 9.3E-01 | 1.0E+00 | NA | **9.0E-03** | 3.7E-01 |  |
| Oleosin 16 kDa-like (LOC123185892), mRNA | 9.6E-01 | 9.9E-01 | **1.8E-02** | 1.0E+00 | 9.4E-01 | **6.8E-03** | 1.0E+00 |  |
| UDP-glucosyltransferase 29-like (LOC123040678), mRNA | 9.9E-01 | 2.3E-01 | **1.0E-03** | **1.6E-02** | 7.6E-01 | **3.8E-02** | 7.6E-01 |  |
| Embryonic protein DC-8-like (LOC123053345), mRNA | 9.8E-01 | **1.1E-02** | 3.6E-01 | 1.0E+00 | 6.9E-01 | 9.9E-01 | 1.0E+00 |  |
| HVA22-like protein k (LOC123044511), mRNA | 9.9E-01 | **4.6E-02** | 9.3E-01 | 1.0E+00 | 9.5E-01 | **3.9E-03** | 1.0E+00 |  |
| Late embryogenesis abundant protein D-34-like (LOC123107821), mRNA | 9.5E-01 | **1.2E-02** | 3.5E-01 | 1.0E+00 | 2.9E-01 | 6.1E-01 | 1.0E+00 |  |
| Non-functional NADPH-dependent codeinone reductase 2-like (LOC119359551), mRNA | 9.9E-01 | **2.1E-03** | 8.1E-01 | 1.0E+00 | 9.3E-01 | 2.3E-01 | 1.0E+00 |  |
| Peroxygenase-like (LOC123041629), mRNA | 8.5E-01 | **2.1E-06** | **9.3E-03** | 1.0E+00 | 6.7E-01 | **3.7E-03** | 9.3E-02 |  |
| Probable aquaporin TIP3-2 (LOC123041656), mRNA | 9.9E-01 | **4.0E-02** | 9.4E-01 | 1.0E+00 | 9.9E-01 | 2.7E-01 | 1.0E+00 |  |
| Oleosin 16 kDa (LOC123046418), mRNA | 9.9E-01 | 7.9E-02 | **2.9E-03** | 1.0E+00 | 8.4E-01 | 9.4E-02 | 1.0E+00 |  |
| Anthocyanidin reductase ((2S)-flavan-3-ol-forming)-like (LOC123046886), mRNA | 9.9E-01 | **7.3E-03** | **3.4E-02** | 1.0E+00 | 9.1E-01 | **4.6E-07** | 1.0E+00 |  |
| Cytochrome P450 89A2-like (LOC119368808), mRNA | 9.4E-01 | **1.9E-02** | NA | 1.0E+00 | NA | 5.1E-02 | 1.0E+00 |  |
| Acetylserotonin O-methyltransferase 3-like (LOC123186559), mRNA | 3.2E-01 | **3.4E-04** | NA | 1.0E+00 | NA | **3.9E-02** | 1.9E-01 |  |
| Clathrin light chain 1-like (LOC123047422), mRNA | 9.4E-01 | **4.6E-02** | 9.7E-01 | 1.0E+00 | 9.2E-01 | 7.8E-01 | 1.0E+00 |  |
| Uncharacterized LOC123060648 (LOC123060648), mRNA | 8.0E-01 | **4.3E-04** | 9.9E-01 | 1.0E+00 | 9.3E-01 | 9.1E-01 | 9.6E-01 |  |
| Calmodulin (CaM6-3A) mRNA, complete cds | 9.9E-01 | **1.7E-02** | 9.3E-01 | 1.0E+00 | 6.9E-01 | **1.6E-02** | **4.3E-03** |  |
| Protein ALP1-like (LOC119268138), transcript variant X1, mRNA | 9.9E-01 | **4.4E-02** | 6.7E-01 | 1.0E+00 | 1.9E-01 | 2.4E-01 | 1.0E+00 |  |
| Uncharacterized LOC123058401 (LOC123058401), mRNA | 9.0E-01 | **1.8E-03** | 9.7E-01 | 1.0E+00 | 6.6E-01 | 3.7E-01 | 1.0E+00 |  |
| Salt tolerant correlative protein mRNA, complete cds | 9.9E-01 | **6.7E-08** | **6.8E-03** | 1.6E-01 | 9.9E-01 | 9.8E-01 | **6.5E-04** |  |
| Cold-shock protein CS120 (LOC123145839), mRNA | 9.9E-01 | **2.0E-02** | 6.2E-01 | 1.0E+00 | 6.3E-01 | 5.7E-02 | 1.0E+00 |  |
| Farnesyl pyrophosphate synthase-like (LOC123061804), transcript variant X3, mRNA | 9.9E-01 | **3.3E-02** | 9.0E-01 | 1.0E+00 | 9.3E-01 | 1.2E-01 | 1.0E+00 |  |
| Oil body-associated protein 2A-like (LOC123058513), mRNA | 9.9E-01 | **5.4E-03** | 6.2E-01 | 1.0E+00 | 6.9E-01 | 9.7E-01 | 1.0E+00 |  |
| Homocysteine S-methyltransferase 4-like (LOC123062208), mRNA | 9.9E-01 | **4.8E-02** | 1.7E-01 | 1.0E+00 | 5.4E-01 | **1.8E-02** | 1.0E+00 |  |
| Protein translation factor SUI1 homolog (LOC100682465), mRNA | 9.9E-01 | **2.5E-02** | 9.1E-01 | 1.0E+00 | **9.3E-03** | **1.3E-02** | 1.0E+00 |  |
| 60S ribosomal protein L10a-like (LOC123062677), mRNA | 9.9E-01 | **4.4E-02** | 9.4E-01 | 1.0E+00 | 8.3E-01 | 4.5E-01 | 7.6E-01 |  |
| Spermatogenesis-associated protein 20-like (LOC119270282), mRNA | 9.9E-01 | **4.0E-02** | 9.7E-01 | 1.0E+00 | 9.7E-01 | 1.5E-01 | 1.0E+00 |  |
| Wheatwin-1 (LOC123063611), mRNA | 9.9E-01 | **7.9E-05** | 9.1E-01 | **4.6E-02** | 9.4E-01 | 2.4E-01 | 2.9E-01 |  |
| 17.5 kDa class II heat shock protein-like (LOC123065002), mRNA | 9.9E-01 | **2.8E-02** | 9.7E-01 | 1.0E+00 | 9.3E-01 | **7.4E-03** | 1.0E+00 |  |
| Uncharacterized LOC123065109 (LOC123065109), mRNA | 9.8E-01 | **4.8E-02** | 9.7E-01 | 1.0E+00 | 9.9E-01 | 2.3E-01 | 1.0E+00 |  |
| Uncharacterized LOC123069245 (LOC123069245), mRNA | 9.5E-01 | **4.0E-02** | 1.0E+00 | 1.0E+00 | 9.9E-01 | 7.3E-02 | 9.9E-01 |  |
| Late embryogenesis abundant protein Lea14-A-like (LOC123069320), mRNA | 9.9E-01 | **1.2E-03** | 1.0E+00 | 1.0E+00 | 8.2E-01 | **9.7E-05** | 1.0E+00 |  |
| Embryonic protein DC-8 (LOC543220), mRNA | 9.9E-01 | **5.7E-03** | 3.8E-01 | 1.0E+00 | 4.1E-01 | **3.2E-04** | 1.0E+00 |  |
| Bifunctional 3-dehydroquinate dehydratase/shikimate dehydrogenase, chloroplastic-like (LOC123069867), transcript variant X3, mRNA | 9.9E-01 | **1.6E-02** | 5.0E-01 | 1.0E+00 | 9.9E-01 | 4.4E-01 | 9.6E-01 |  |
| Uncharacterized LOC123065488 (LOC123065488), mRNA | 9.9E-01 | **1.8E-06** | 8.9E-01 | 1.0E+00 | 6.5E-01 | 2.0E-01 | 7.8E-01 |  |
| Gallate 1-beta-glucosyltransferase 84A23-like (LOC123065579), mRNA | 9.8E-01 | **4.0E-02** | **8.4E-04** | 1.0E+00 | 7.1E-01 | **1.3E-04** | **4.3E-03** |  |
| Universal stress protein PHOS34-like (LOC123107837), transcript variant X1, mRNA | 8.6E-01 | **4.9E-04** | **4.1E-02** | 1.0E+00 | 5.7E-01 | **7.7E-03** | **5.1E-03** |  |
| Uncharacterized LOC123072136 (LOC123072136), mRNA | 9.9E-01 | **1.3E-02** | 9.0E-01 | 1.0E+00 | 9.5E-01 | 9.8E-01 | 1.0E+00 |  |
| Taxi-I mRNA for xylanase inhibitor TAXI-I, complete cds | 9.9E-01 | **3.4E-04** | 9.6E-01 | 1.0E+00 | 8.3E-01 | **2.3E-02** | 1.0E+00 |  |
| 40S ribosomal protein S10-1-like (LOC123072688), mRNA | 1.0E+00 | **7.1E-03** | 9.1E-01 | 1.0E+00 | 9.7E-01 | **8.8E-03** | 1.0E+00 |  |
| Cytochrome b5-like (LOC119280312), mRNA | 9.9E-01 | **4.5E-02** | 9.7E-01 | 1.0E+00 | 9.6E-01 | 2.8E-01 | 1.0E+00 |  |
| Late embryogenesis abundant protein D-34-like (LOC123084873), mRNA | 8.2E-01 | 2.9E-01 | **1.2E-02** | 1.0E+00 | 3.0E-01 | 3.0E-01 | **6.6E-04** |  |
| Heat shock cognate 70 kDa protein-like (LOC123085462), mRNA | 9.6E-01 | **1.9E-02** | 7.0E-01 | 1.0E+00 | 9.1E-01 | **2.6E-02** | 8.1E-01 |  |
| Uncharacterized protein Mb0911c-like (LOC123081835), mRNA | 9.9E-01 | **2.4E-03** | 9.7E-01 | 1.0E+00 | 8.7E-01 | **3.9E-07** | 1.0E+00 |  |
| Late embryogenesis abundant protein 1-like (LOC123081904), mRNA | 1.8E-01 | **6.0E-04** | 1.5E-01 | 1.0E+00 | 1.5E-01 | 1.9E-01 | 6.1E-01 |  |
| Vicilin-like seed storage protein At2g28490 (LOC123085798), mRNA | 3.8E-01 | **3.8E-07** | 9.7E-01 | 1.0E+00 | 7.6E-01 | **2.7E-02** | 1.0E+00 |  |
| Dehydrin DHN4-like (LOC119286757), transcript variant X3, mRNA | 9.6E-01 | **7.6E-03** | 6.6E-01 | 1.0E+00 | 6.4E-01 | 1.2E-01 | 1.0E+00 |  |
| Uncharacterized LOC100682464 (LOC100682464), mRNA | 8.5E-01 | **2.6E-02** | 8.2E-01 | 1.0E+00 | 4.8E-01 | 9.3E-01 | 1.0E+00 |  |
| PM19-A1 gene, complete cds | 9.3E-01 | **1.2E-04** | 9.7E-01 | 1.0E+00 | 6.4E-01 | 1.5E-01 | 1.0E+00 |  |
| PM19-A2 gene, complete cds | 9.6E-01 | **2.4E-03** | 4.5E-01 | 1.0E+00 | 9.0E-01 | 6.9E-01 | 9.7E-01 |  |
| Epoxide hydrolase A-like (LOC123121137), mRNA | 9.9E-01 | **3.6E-03** | 9.9E-01 | 1.0E+00 | 9.5E-01 | 1.8E-01 | 1.0E+00 |  |
| Histone H1-like (LOC119287984), mRNA | 2.1E-01 | **2.1E-07** | 7.5E-01 | 1.0E+00 | 7.6E-01 | 4.1E-01 | **1.6E-02** |  |
| ABC transporter F family member 1-like (LOC123090461), mRNA | 7.1E-01 | **1.9E-02** | **9.4E-04** | 1.0E+00 | 6.9E-01 | **1.7E-05** | **4.3E-03** |  |
| 63 kDa globulin-like protein (LOC123090599), mRNA | 9.9E-01 | **4.0E-04** | 9.7E-01 | **2.5E-03** | 9.3E-01 | 6.5E-01 | 1.0E+00 |  |
| 63 kDa globulin-like protein (LOC123090601), mRNA | 9.9E-01 | **7.0E-10** | 1.7E-01 | **4.3E-02** | 9.9E-01 | 5.1E-01 | 2.2E-01 |  |
| Heavy metal-associated isoprenylated plant protein 7-like (LOC123094567), mRNA | 9.9E-01 | **7.9E-06** | 8.8E-01 | 1.0E+00 | 9.5E-01 | **1.9E-05** | 1.0E+00 |  |
| Sphinganine C4-monooxygenase 1-like (LOC123094575), mRNA | 1.0E+00 | **8.3E-03** | NA | 1.0E+00 | NA | 6.8E-01 | 1.0E+00 |  |
| NAP1-related protein 2-like (LOC123137142), mRNA | 9.9E-01 | **7.0E-03** | 1.0E+00 | **2.5E-02** | 8.7E-01 | **4.0E-03** | 1.0E+00 |  |
| Late embryogenesis abundant protein 1-like (LOC119293714), mRNA | 8.1E-01 | **5.8E-04** | 9.3E-01 | 1.0E+00 | 7.9E-01 | 1.8E-01 | 1.0E+00 |  |
| Uncharacterized LOC123094952 (LOC123094952), mRNA | 9.5E-01 | **4.6E-02** | 9.7E-01 | 1.0E+00 | 4.3E-01 | **1.2E-03** | 1.0E+00 |  |
| PDW 291 small heat shock protein 26 (sHSP26) gene, complete cds | 9.1E-01 | **6.7E-08** | NA | 1.0E+00 | NA | 2.3E-01 | 8.2E-01 |  |
| NHP2-like protein 1 (LOC123092812), mRNA | 9.1E-01 | **1.9E-02** | 9.7E-01 | 1.0E+00 | 9.8E-01 | **1.8E-03** | 1.0E+00 |  |
| Beta-amylase 2, chloroplastic-like (LOC119292021), mRNA | 9.9E-01 | **4.1E-04** | 4.1E-01 | 1.0E+00 | 9.9E-01 | **1.1E-02** | 1.0E+00 |  |
| Plasma membrane ATPase 1-like (LOC123101933), transcript variant X1, mRNA | 9.6E-01 | **2.8E-02** | **3.4E-02** | 1.0E+00 | 9.0E-01 | 3.5E-01 | 1.0E+00 |  |
| Glycine-rich RNA-binding protein blt801-like (LOC123102010), mRNA | 6.1E-02 | 9.7E-01 | **2.9E-02** | 1.0E+00 | **1.3E-02** | 1.9E-01 | 6.5E-01 |  |
| Phosphoenolpyruvate carboxylase 2-like (LOC123103489), mRNA | 9.9E-01 | 7.0E-01 | **3.0E-02** | 1.0E+00 | 8.2E-01 | **9.5E-04** | 2.8E-01 |  |
| Isochorismate synthase 2, chloroplastic-like (LOC119301387), transcript variant X1, mRNA | 9.9E-01 | **4.4E-02** | 9.8E-01 | 1.0E+00 | 9.9E-01 | **2.7E-02** | 1.0E+00 |  |
| Succinate dehydrogenase [ubiquinone] iron-sulfur subunit 2, mitochondrial-like (LOC123106994), mRNA | 9.9E-01 | 3.7E-01 | **3.0E-02** | 1.0E+00 | 9.4E-01 | 2.1E-01 | **2.9E-02** |  |
| 17.4 kDa class I heat shock protein-like (LOC123159544), mRNA | 9.9E-01 | 7.6E-01 | **3.6E-02** | 1.0E+00 | 7.6E-01 | 6.1E-01 | 1.0E+00 |  |
| Defensin Ec-AMP-D2-like (LOC123105798), mRNA | 9.9E-01 | **3.0E-04** | 5.0E-01 | 1.0E+00 | 9.1E-01 | 1.6E-01 | 1.0E+00 |  |
| Late embryogenesis abundant protein 3-like (LOC123115469), mRNA | 7.0E-01 | **1.6E-03** | 9.3E-01 | 1.0E+00 | 6.9E-01 | 1.2E-01 | 1.0E+00 |  |
| Outer membrane channel protein OEP16-2 (OEP16-2) gene, complete cds | 8.4E-01 | **1.6E-02** | 4.7E-01 | 1.0E+00 | 2.5E-01 | 1.8E-01 | 1.0E+00 |  |
| Lipid transfer protein 1 precursor (LTP1500) gene, promoter region, exons 1 and 2, and complete cds | 9.9E-01 | **1.5E-03** | 6.2E-01 | 1.0E+00 | 9.1E-01 | **1.6E-03** | 1.0E+00 |  |
| Oleosin 16 kDa-like (LOC123116071), mRNA | 9.1E-01 | **6.2E-04** | 9.8E-01 | 1.0E+00 | 9.9E-01 | **1.8E-02** | 1.0E+00 |  |
| 16.9 kDa class I heat shock protein 1-like (LOC119305694), mRNA | 9.4E-01 | **5.0E-16** | 6.2E-01 | 1.0E+00 | 9.3E-01 | **6.3E-04** | 9.9E-01 |  |
| Cysteine proteinase 1-like (LOC119311326), mRNA | 9.9E-01 | **1.1E-02** | 8.9E-01 | 1.0E+00 | 8.6E-01 | **1.9E-04** | 1.0E+00 |  |
| PAPhy_a3 gene for Purple acid phosphatase phytase, strain Tri18523 | 9.9E-01 | **1.6E-02** | 8.6E-01 | 1.0E+00 | 8.4E-01 | **5.9E-03** | 1.0E+00 |  |
| Probable prefoldin subunit 4 (LOC123113248), mRNA | 9.9E-01 | **3.0E-02** | 9.9E-01 | 1.0E+00 | 9.9E-01 | **1.7E-02** | 1.0E+00 |  |
| Cupincin-like (LOC119306383), mRNA | 4.8E-01 | **4.5E-09** | 9.7E-01 | 1.0E+00 | 9.3E-01 | **2.8E-03** | 1.0E+00 |  |
| GEM-like protein 5 (LOC123114069), mRNA | 9.4E-01 | **1.9E-02** | NA | 1.0E+00 | NA | 2.0E-01 | 8.2E-01 |  |
| 11-beta-hydroxysteroid dehydrogenase B-like (LOC119306749), mRNA | 1.1E-01 | 1.5E-01 | **9.9E-03** | 1.0E+00 | 1.3E-01 | 2.6E-01 | **8.9E-03** |  |
| Membrane protein PM19L-like (LOC123117892), mRNA | 9.3E-01 | **6.7E-08** | 5.3E-01 | 1.0E+00 | 9.9E-01 | 1.8E-01 | 1.0E+00 |  |
| Membrane protein PM19L-like (LOC123117893), mRNA | 9.9E-01 | **1.6E-02** | 8.2E-01 | 1.0E+00 | 8.0E-01 | 1.0E-01 | 1.0E+00 |  |
| Xylanase inhibitor protein 1-like (LOC119314698), mRNA | 9.9E-01 | **8.9E-03** | 7.3E-01 | 1.0E+00 | 9.3E-01 | 3.6E-01 | 7.8E-01 |  |
| Molybdopterin synthase sulfur carrier subunit-like (LOC123127918), mRNA | 9.9E-01 | **4.0E-02** | 8.5E-01 | 1.0E+00 | 9.9E-01 | 1.0E-01 | 1.0E+00 |  |
| Dehydrin DHN4-like (LOC119318248), mRNA | 9.9E-01 | **5.7E-04** | **2.7E-02** | 1.0E+00 | 9.9E-01 | 6.9E-01 | 1.0E+00 |  |
| Xylanase inhibitor protein 1-like (LOC123133608), mRNA | 9.1E-01 | **4.8E-02** | 9.7E-01 | 1.0E+00 | NA | **3.7E-06** | 1.0E+00 |  |
| Peroxisomal fatty acid beta-oxidation multifunctional protein-like (LOC119325551), mRNA | 2.2E-01 | **1.6E-02** | NA | 1.0E+00 | NA | **3.0E-04** | 1.0E+00 |  |
| Desmethyl-deoxy-podophyllotoxin synthase-like (LOC123133937), mRNA | 9.9E-01 | **4.0E-02** | 9.0E-01 | 1.0E+00 | 7.6E-01 | 5.2E-02 | 1.0E+00 |  |
| Probable tyrosine-protein phosphatase DSP2 (LOC119323088), mRNA | 7.6E-01 | **1.2E-02** | **8.4E-04** | 1.0E+00 | 9.7E-01 | 1.1E-01 | 1.0E+00 |  |
| V-type proton ATPase 16 kDa proteolipid subunit-like (LOC123103600), mRNA | 8.4E-01 | **2.6E-05** | **8.4E-05** | 1.0E+00 | 9.4E-01 | **1.1E-02** | **7.4E-03** |  |
| Dehydrin DHN4 (LOC543296), mRNA | 9.9E-01 | **1.9E-05** | 2.6E-01 | 1.0E+00 | 7.4E-01 | **6.7E-03** | 1.0E+00 |  |
| Salt-induced YSK2 dehydrin 2 (DHN2) mRNA, complete cds | 9.9E-01 | 5.8E-01 | **7.4E-04** | 1.0E+00 | 9.8E-01 | 3.5E-01 | 1.0E+00 |  |
| Late embryogenesis abundant protein 6-like (LOC119330636), mRNA | 9.8E-01 | **3.6E-03** | 6.4E-01 | 1.0E+00 | 9.5E-01 | **1.8E-03** | 1.0E+00 |  |
| Bidirectional sugar transporter SWEET15-like (LOC119337093), mRNA | 6.8E-02 | **4.2E-02** | NA | 1.0E+00 | NA | **1.9E-02** | 9.6E-01 |  |
| Bidirectional sugar transporter SWEET15-like (LOC119329948), mRNA | 7.6E-01 | **1.2E-02** | 8.6E-01 | 1.0E+00 | 7.6E-01 | **4.6E-03** | 8.8E-01 |  |
| Protein DETOXIFICATION 16-like (LOC123150698), transcript variant X1, mRNA | 9.6E-01 | **2.0E-02** | NA | 1.0E+00 | NA | 6.5E-02 | 1.0E+00 |  |
| Calmodulin (CaM13-7A) mRNA, complete cds | 9.9E-01 | **4.8E-02** | 9.7E-01 | 1.0E+00 | 9.9E-01 | 6.6E-02 | 1.0E+00 |  |
| Chitinase 1-like (LOC123148127), mRNA | 9.9E-01 | **8.6E-04** | 4.7E-01 | 1.0E+00 | 9.1E-01 | **2.4E-02** | 1.0E+00 |  |
| Catalase 3 (Cat3-A1) gene, Cat3-A1b allele, complete cds | 9.9E-01 | **5.6E-04** | 9.9E-01 | 1.0E+00 | 9.1E-01 | **3.5E-02** | 1.2E-01 |  |
| 28S ribosomal RNA (LOC123140494), rRNA | 9.8E-01 | **3.7E-04** | 9.9E-01 | 1.0E+00 | 9.3E-01 | **1.2E-03** | 1.0E+00 |  |
| Bidirectional sugar transporter SWEET15-like (LOC123157400), mRNA | 9.6E-01 | **2.2E-02** | NA | 1.0E+00 | NA | 6.0E-02 | 9.3E-01 |  |
| Late embryogenesis abundant protein 19-like (LOC119368216), mRNA | 9.8E-01 | 4.2E-01 | **3.5E-02** | 1.0E+00 | 8.3E-01 | 4.3E-01 | 2.4E-01 |  |
| Protein LE25-like (LOC123160757), mRNA | 4.5E-01 | **1.3E-02** | 6.2E-01 | 1.0E+00 | 1.3E-01 | **2.2E-02** | 1.0E+00 |  |
| 60S acidic ribosomal protein P0 (LOC123162195), mRNA | 9.9E-01 | **4.9E-02** | 1.0E+00 | 1.0E+00 | 8.9E-01 | **7.4E-04** | 1.0E+00 |  |
| Probable calcium-binding protein CML7 (LOC119341174), mRNA | 9.8E-01 | **3.0E-02** | 9.7E-01 | 1.0E+00 | 9.8E-01 | **3.3E-05** | 1.0E+00 |  |
| Defensin Tm-AMP-D1.2-like (LOC123157903), mRNA | 9.9E-01 | **8.6E-04** | 9.7E-01 | 1.0E+00 | 8.9E-01 | **4.9E-02** | 7.9E-01 |  |
| Late embryogenesis abundant protein 31-like (LOC123158928), mRNA | 9.6E-01 | **2.0E-02** | 9.6E-01 | 1.0E+00 | 8.9E-01 | **3.0E-02** | 1.0E+00 |  |
| 40S ribosomal protein S24-1 (LOC123156961), mRNA | 9.1E-01 | **2.9E-02** | 9.8E-01 | 1.0E+00 | 4.1E-01 | 6.3E-01 | 1.0E+00 |  |
| 40S ribosomal protein S25-2 (LOC123158982), mRNA | 9.8E-01 | **1.3E-03** | 9.7E-01 | 1.0E+00 | 5.4E-01 | **1.9E-02** | 1.0E+00 |  |
| Non-specific lipid-transfer protein 1-like (LOC123158491), transcript variant X1, mRNA | **2.1E-03** | NA | NA | 1.0E+00 | NA | 3.0E-01 | 1.0E+00 |  |
| Voucher H3288 clone 2 alcohol dehydrogenase (adh1) gene | **1.5E-02** | 7.2E-01 | 1.0E+00 | 1.0E+00 | 6.3E-01 | 9.9E-01 | 8.6E-01 |  |
| 28S ribosomal RNA (LOC123152675), rRNA | **4.6E-02** | 7.7E-01 | 8.0E-01 | 1.0E+00 | 8.1E-01 | 1.9E-01 | 1.0E+00 |  |
| Cysteine proteinase inhibitor 8-like (LOC123187619), mRNA | 3.5E-01 | **5.8E-03** | 8.9E-01 | 1.0E+00 | 2.8E-01 | **2.5E-02** | 9.6E-01 |  |
| Probable cysteine protease RD19D (LOC123188105), mRNA | 7.0E-02 | **9.0E-03** | 9.4E-01 | 1.0E+00 | 7.9E-01 | **9.4E-03** | 1.0E+00 |  |
| Protein translation factor SUI1 homolog (LOC123188402), mRNA | **4.6E-02** | 5.4E-01 | 8.1E-01 | 1.0E+00 | 6.2E-02 | 3.9E-01 | 1.0E+00 |  |
| Gamma gliadin-B1, gamma gliadin-B2, delta gliadin-B1, gamma gliadin-B4, gamma gliadin-B6, omega gliadin-B3, omega gliadin-B6, LMW-B2, and LMW-B3 genes, complete cds | **3.4E-03** | 9.8E-01 | 1.0E+00 | 1.0E+00 | 3.7E-01 | 9.6E-01 | 1.0E+00 |  |
| Glycerophosphodiester phosphodiesterase GDPD1, chloroplastic-like (LOC123071179), transcript variant X1, mRNA | **4.9E-04** | 3.8E-01 | 8.4E-01 | 1.0E+00 | 9.7E-01 | 7.6E-01 | 5.7E-01 |  |
| Luminal-binding protein 2 (LOC119319558), mRNA | **2.2E-02** | NA | NA | 1.0E+00 | NA | **2.8E-03** | 9.6E-01 |  |
| Protein GDAP2 homolog (LOC123137672), mRNA | **7.1E-04** | 8.7E-02 | 6.1E-01 | 1.0E+00 | 9.3E-01 | **3.8E-04** | 9.2E-01 |  |
| Putative transferase At4g12130, mitochondrial (LOC119347614), mRNA | **2.6E-02** | NA | NA | 1.0E+00 | NA | **2.0E-03** | 3.3E-01 |  |
| Late embryogenesis abundant protein 31-like (LOC123151325), mRNA | **5.8E-03** | 4.6E-01 | 9.5E-01 | 1.0E+00 | 4.3E-01 | 3.5E-01 | 1.0E+00 |  |
| Polynucleotide 5'-hydroxyl-kinase NOL9-like (LOC123068941), transcript variant X2, mRNA | 7.1E-01 | 7.0E-01 | 8.8E-01 | **2.8E-02** | 8.3E-01 | 4.5E-01 | 1.0E+00 |  |
| Peroxidase 55-like (LOC123162898), mRNA | NA | **4.3E-02** | NA | NA | NA | **1.7E-03** | NA |  |

| **Supplementary Table S3.** The effect of inoculation with a consortium of four beneficial bacteria and stress treatments on the enzymatic antioxidant (A), non-enzymatic components of the antioxidant system and marker of oxidative stress (B) antioxidant in wheat grain. | | | | | | | | |
| --- | --- | --- | --- | --- | --- | --- | --- | --- |
| Parameter | Unit |  | Salinity | | Drought | | Non-stress | |
| *(A)* |  |  | Co | BC | Co | BC | Co | BC |
| APX | µmol min^-1^ mg^-1^ protein |  | 0.38 ± 0.03 b | 0.36 ± 0.04 b | 0.42 ± 0.03 b | 0.59 ± 0.04 a | 0.15 ± 0.04 c | 0.21 ± 0.03 c |
| SOD | µmol min^-1^ mg^-1^ protein |  | 4.20 ± 0.36 b | 3.67 ± 0.23 b | 3.77 ± 0.35 b | 4.77 ± 0.21 a | 1.08 ± 0.11 c | 1.34 ± 0.19 c |
| CAT | µmol min^-1^ mg^-1^ protein |  | 1.12 ± 0.03 b | 0.87 ± 0.03 bc | 1.24 ± 0.03 b | 1.90 ± 0.03 a | 0.65 ± 0.03 c | 0.73 ± 0.03 c |
| POX | µmol min^-1^ mg^-1^ protein |  | 0.21 ± 0.03 c | 0.52 ± 0.03 b | 0.30 ± 0.04 c | 0.78 ± 0.09 a | 0.11 ± 0.02 d | 0.27 ± 0.04 c |
| MDHAR | nmolmin^-1^ mg^-1^ protein |  | 1.28 ± 0.14 c | 2.12 ± 0.25 a | 0.51 ± 0.06 d | 1.65 ± 0.12 b | 0.23 ± 0.02 e | 0.33 ± 0.04 de |
| DHAR | nmolmin^-1^ mg^-1^ protein |  | 0.39 ± 0.04 b | 0.70 ± 0.06 a | 0.28 ± 0.04 b | 0.61 ± 0.07 a | 0.02 ± 0.00 c | 0.07 ± 0.00 c |
| GR | nmol min^-1^ mg^-1^ protein |  | 0.30 ± 0.05 d | 1.15 ± 0.13 b | 0.61 ± 0.07 c | 1.47 ± 0.17 a | 0.03 ± 0.00 e | 0.04 ± 0.00 e |
| *(B)* |  |  |  |  |  |  |  |  |
| Grx | nmol g ^-1^ DW |  | 6.07 ± 0.53 b | 5.56 ± 0.46 b | 6.07 ± 0.37 b | 7.44 ± 0.55 a | 2.69 ± 0.34 c | 2.44 ± 0.29 c |
| Trx | nmol g ^-1^ DW |  | 4.69 ± 0.28 c | 7.15 ± 0.81 b | 3.78 ± 0.44 d | 7.85 ± 0.56 a | 3.08 ± 0.36 e | 4.05 ± 0.41cd |
| MDA | nmol g ^-1^ DW |  | 7.58 ± 0.66 a | 5.83 ± 0.49 b | 7.76 ± 0.81 a | 5.47 ± 0.44 b | 3.79 ± 0.36 c | 3.67 ± 0.41 c |
| GSH | nmol g ^-1^ DW |  | 0.55 ± 0.06 c | 2.01 ± 0.31 a | 0.53 ± 0.38 c | 1.50 ± 0.16 b | 0.07 ± 0.00 d | 0.12 ± 0.02 d |
| Anthocyanidin | mg g^−1^ DW |  | 2.31 ± 0.33 b | 2.75 ± 0.39 b | 2.21 ± 0.16 b | 4.40 ± 0.26 a | 1.49 ± 0.17 c | 1.54 ± 0.14 c |
| ACNs | mg g^−1^ DW |  | 3.63 ± 0.30 b | 4.02 ± 0.26 b | 3.64 ± 0.38 b | 6.74 ± 0.46 a | 1.82 ± 0.11 c | 2.10 ± 0.25 c |
| Prxs | mol H_2_O_2_ mol Prx min^−1^ |  | 2.11 ± 0.16 c | 2.87 ± 0.33 b | 1.58 ± 0.14 d | 3.74 ± 0.30 a | 1.10 ± 0.09 e | 1.33 ± 0.14 de |
| ASC-AT | nmol g ^-1^ DW |  | 0.83 ± 0.09 b | 1.63 ± 0.20 a | 1.14 ± 0.13 b | 1.53 ± 0.22 a | 0.41 ± 0.05 c | 0.40 ± 0.04 c |
| GPx | nmol min^-1^ mg^-1^ protein |  | 1.28 ± 0.10 b | 2.29 ± 0.22 a | 1.48 ± 0.19 b | 2.52 ± 0.31 a | 0.27 ± 0.03 c | 0.44 ± 0.05 c |
| Means (± standard deviation; n = 6) in each parameter followed by similar letter(s) are not significantly different at 5% probability level (Tukey test).  Co: No fertilization (control); BC: Biofertilizer consortium of four PGPB strains.  CAT: Catalase; POX: Peroxidase; SOD: Superoxide dismutase; MDHAR: Monodehydroascorbate reductase; DHAR: Dehydroascorbate reductase; APX: Ascorbate peroxidase; GR: Glutathione reductase; GSH: Glutathione; MDA: Malondialdehyde; Trx: Thioredoxin; Prx: peroxiredoxin; Grx: Glutaredoxin; ACNs:  Anthocyanins; ASC-AT: ASC amino acid transporter-2; GPx: Glutathione peroxidase. | | | | | | | | |
